# Domesticated bacterial origin genes regulate homeostasis of the insect gut microbiome and empower adaptation

**DOI:** 10.1002/imt2.70179

**Published:** 2026-10-15

**Authors:** Zhenjia Zhang, Yubo Zhang, Kun Zhao, Guangyuan Cheng, Zhihao Lu, Dongju Wang, Yiqun Liu, Jianguo Wu, Liying Wang, Lisheng Zhang, Xinhai Ye, Li Yu, Wei Zhang, Yi Li

**Affiliations:** ^1^ State Key Laboratory of Agricultural and Forestry Biosecurity, Vector‐borne Virus Research Center Fujian Agriculture and Forestry University Fuzhou China; ^2^ State Key Laboratory of Gene Function and Modulation Research, School of Life Sciences Peking University Beijing China; ^3^ College of Grassland Science and Technology China Agricultural University Beijing China; ^4^ The State Key Laboratory of Membrane Biology Tsinghua University‐Peking University Joint Centre for Life Sciences, Beijing Frontier Research Center for Biological Structure, School of Life Sciences, Tsinghua University Beijing China; ^5^ National Center for Protein Science and Core Facilities at School of Life Sciences Peking University Beijing China; ^6^ Key Laboratory of Natural Enemy Insects of Ministry of Agriculture and Rural Affairs, State Key Laboratory for Biology of Plant Diseases and Insect Pests Institute of Plant Protection, Chinese Academy of Agricultural Sciences Beijing China; ^7^ College of Advanced Agriculture Sciences Zhejiang A&F University Hangzhou China; ^8^ Peking‐Tsinghua Center for Life Sciences, Academy for Advanced Interdisciplinary Studies Peking University Beijing China; ^9^ Institute of Ecology Peking University Beijing China; ^10^ Peking University Chengdu Academy for Advanced Interdisciplinary Biotechnologies Chengdu China; ^11^ Medog Biodiversity Observation and Research Station of Xizang Autonomous Region Nyingchi China

**Keywords:** adaptation, cholesterol‐dependent cytolysin, functional domestication and diversification, gut microbiome, Leafhopper

## Abstract

Sap‐sucking insects, represented by the globally distributed leafhoppers, have evolved a wide range of adaptive traits and play multiple ecological roles. However, their successful survival strategies in nature remain poorly understood. To explore the adaptive mechanisms of sap‐sucking insects, we established a research system using three representative leafhopper species from the same family and found that they have acquired multiple cholesterol‐dependent cytolysins (*CDCs*) derived from gram‐positive bacterial genes. Furthermore, we demonstrated that these *CDCs* have been domesticated and co‐evolved with leafhoppers, ultimately participating in various life activities of the host. Specifically, some *CDCs* exhibit high cytotoxicity, are specifically expressed in the egg serosa, and affect the feeding behaviors of leafhopper predators; others show non‐cytotoxic activity, are specifically expressed in the gut, and may regulate leafhopper growth and development by potentially maintaining the homeostasis of the leafhopper gut microbiome. This study uncovers leafhoppers as a paradigm for understanding how organisms can borrow microbial innovations to address ecological challenges and thrive as agricultural pests. It provides a comprehensive perspective to bridge the genotypes of acquired genes with the phenotypes of adaptive traits and demonstrates how homologous genes from donor organisms undergo functional differentiation in recipient hosts, enabling them to exploit new ecological niches. By decoding these mechanisms, we not only gain fundamental insights into evolutionary processes but also develop powerful strategies for sustainable pest management.

## Highlights

- Leafhoppers acquired cholesterol‐dependent cytolysin genes from gram‐positive bacteria by horizontal gene transfer.
- Conserved and toxic cytolysins are specifically expressed in leafhopper egg serosa as a defense weapon against natural enemies.
- Nontoxic cytolysins gain novel functions, are specifically expressed in the gut, and may contribute to leafhopper growth and development by potentially maintaining gut microbiome homeostasis.
- Domestication and diversification of exogenous genes improve the fitness of leafhoppers in multiple ways.

## Graphical abstract

Horizontal gene transfer introduced bacterial *CDC* genes into the leafhopper genome, providing genetic material for subsequent evolutionary innovation. Different *CDC* copies diversified at the sequence, structural, and functional levels. RdCDC3 retained its ancestral pore‐forming activity and was recruited for egg defense, whereas RdCDC9 lost its toxicity and may contribute to the regulation of the gut microbial community and host development. These findings highlight the functional diversification and adaptive significance of horizontally acquired genes in leafhoppers.

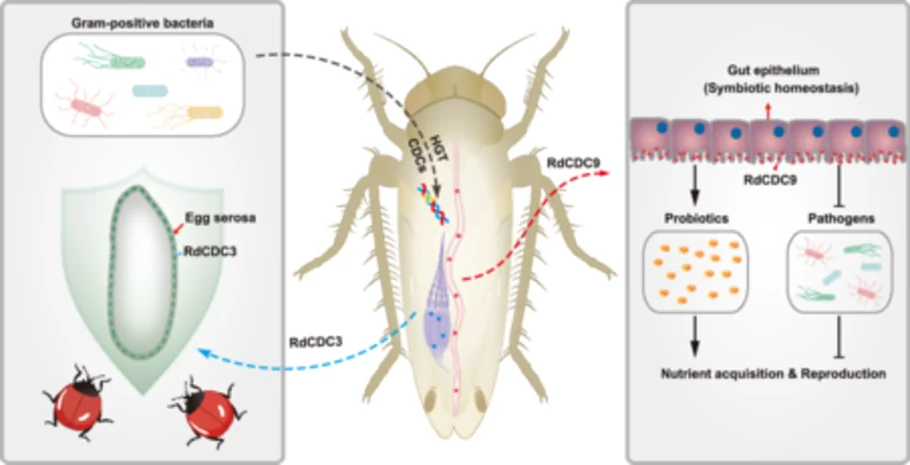

## INTRODUCTION

Sap‐sucking insects feed on the phloem sap of vascular plants with their needle‐like piercing mouthparts. Due to their unique foraging habits and life histories, they harbor complex ecological roles as prey or pollinators and as vectors of pests and plant pathogens, which are vital to ecosystems and require in‐depth understanding to facilitate biodiversity conservation and ecological balance maintenance [1, 2, 3, 4]. For example, the leafhoppers in Cicadellidae, representatives of a vast number of sap‐sucking insects, are widely distributed, with more than 20,000 described species, many of which are important agricultural pests and a primary group of confirmed insect vectors of plant pathogens [5, 6, 7, 8]. As a result, leafhoppers have become an ideal research system for investigating complex interactions among insects, symbionts, plants, and pathogens, yielding research methods and findings to benefit further exploration of their adaptive strategies [7, 8, 9, 10, 11]. As revealed in other insects, it is likely that the leafhoppers have developed specialized pathways to confer resistance to viral, bacterial, and fungal pathogens in response to numerous challenges from the environment, such as natural predators and pathogens [12, 13, 14, 15]. Nevertheless, little is known about how leafhoppers balance defense mechanisms and life history in such complex ecological roles.

*Recilia dorsalis, Nephotettix cincticeps*, and *Nephotettix nigropictus* are three agriculturally important leafhopper species in the family Cicadellidae. They are important pests of rice plants, are widely distributed in Asia, and are commonly found in rice agroecosystems. These three species are also vectors of diverse plant pathogens, including viruses and phytoplasmas, many of which are transmitted in a persistent and propagative manner [7, 8]. These pathogens primarily affect rice and other crops, causing recurrent disease outbreaks and significant agricultural losses. Therefore, understanding the evolutionary trajectories and adaptive mechanisms of these leafhopper species has important ecological implications and may provide insights into crop protection and food security.

Here, based on high‐quality genomes of these three leafhoppers, combined with bioinformatic, molecular, and biochemical information as well as cell biology and insect performance assays, we showed that the genomes of *R. dorsalis, Ne. cincticeps*, and *Ne. nigropictus* harbor multiple horizontally transferred genes encoding cholesterol‐dependent cytolysins (CDCs) of gram‐positive bacterial origin that lyse eukaryotic cells by forming pores in the cell membrane through the binding of cholesterol, the major target cell receptor [16, 17, 18, 19]. We further characterized these *CDC* genes in *R. dorsalis* (*RdCDCs*) and found that they have been domesticated and evolved with functional differentiation, ranging from strong to weak or even no cholesterol affinity and from high to weak or even no cytotoxicity to eukaryotic cells. A comprehensive study of RdCDC3, which has high affinity for cholesterol and high cytotoxicity, showed that it was expressed specifically in the egg serosa and affected the feeding behaviors of leafhopper predators, such as ladybugs. In contrast, RdCDC9, which has no cholesterol affinity or cytotoxicity, underwent positive selection and was expressed specifically in the gut and associated with the maintenance of gut microbiome homeostasis and leafhopper survival.

In summary, our results reveal a historical genome evolutionary event driving acquired adaptive traits in leafhoppers, which, overall, may deepen the understanding of environmental adaptations in the insect world and facilitate the development of novel strategies for the control of these widespread pests and the diseases they transmit. Moreover, leafhoppers and *CDC* genes may serve as a model system for future mechanistic studies of the domestication and evolution of genes in recipient insects.

## RESULTS

### Structural features and evolution of the leafhopper genome

To characterize genomic signatures related to adaptive traits in leafhoppers, we focused on three representative leafhopper species, *R. dorsalis*, *Ne. cincticeps*, and *Ne. nigropictus*, which are widely distributed and have adapted to various environmental conditions. These leafhoppers are particularly prevalent in Asia and the Pacific, where they are commonly found near rice crops (PlantwisePlus Knowledge Bank, CABI International) (Figure 1A). Chromosome‐level reference genomes were assembled by integrating PacBio long reads, Illumina paired‐end reads, and Hi‐C data (Figures 1A and S1; Table S1). Compared with the nearly identical genome sizes and high levels of synteny between the two *Nephotettix* species (788 Mb in *Ne. cincticeps* and 770 Mb in *Ne. nigropictus*) (Figure 1B), *R*. *dorsalis* exhibited a larger genome (1223 Mb), possibly due to the expansion of repetitive sequences, which constituted approximately 60% of the *R. dorsalis* genome (Table S2), and a few chromosomal fusions, fissions and translocations. In contrast, the sex chromosome was conserved between the two genera. *Nephotettix* chromosome 7 and its homologous sequence in *R. dorsalis* (chromosome 9) were identified as X chromosomes because their read coverage was half that of the other autosomes when Illumina reads from males were mapped to the assemblies (Figure 1B,C). Similarly, *R. dorsalis* has more species‐specific genes (3998) than *Ne. cincticeps* (474) and *Ne. nigropictus* (282), likely due to its larger genome and greater number of genes (Figure 1D and Table S2).

**FIGURE 1:**
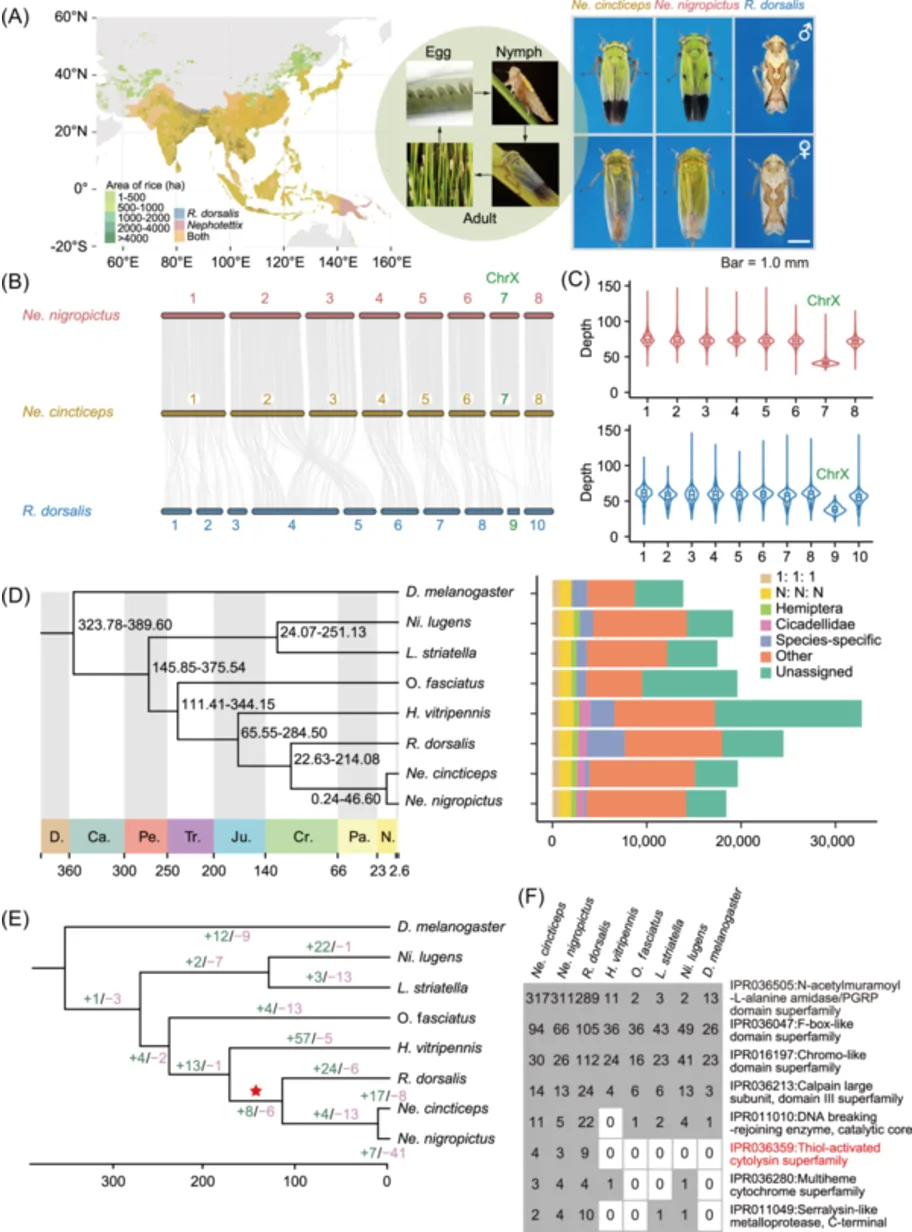
Distribution and comparative genomic analysis of three leafhopper species. (A) Distribution (left), life history (middle), and photographs (right) of three species of leafhoppers. Overlapping regions between records from *Nephotettix* and *R. dorsalis* are shown in yellow, whereas specific regions of *Nephotettix* and *R. dorsalis* are displayed in lilac and blue, respectively. Rice cultivation areas are shown on the map with a green color gradient (data obtained from MapSPAM). Three leafhopper species, *Ne. cincticeps* (right: left), *Ne. nigropictus* (right: middle), and *R. dorsalis* (right: right), were used for genome sequencing and other research. ha: hectare; ♂: male; ♀: female. Bar = 1.0 mm. (B) Collinearity relationships among the three genomes. Syntenic blocks are represented by each line. The sex chromosome (ChrX) is labeled in green. (C) Violin plots summarizing the read depths on each chromosome in male *Ne. nigropictus* and *R. dorsalis* with a window size of 100 kb. (D) Time‐calibrated phylogenetic tree of seven Hemiptera species with an outgroup taxon, *Drosophila melanogaster*. Numbers at the nodes represent the 95% confidence intervals of each species divergence time (million years ago, Mya). The maximum likelihood tree was constructed based on the sequence alignments of 729 single‐copy genes. The histogram shows the number of genes in each orthogroup category. 1:1:1 indicates single‐copy genes; N:N:N indicates orthogroups present in all species; Hemiptera indicates genes present in seven Hemiptera species; Cicadellidae indicates genes present in four Cicadellidae species; Species‐specific indicates species‐specific genes; Other indicates all other types; and Unassigned indicates genes that are not assigned to orthogroups. D, Ca, Pe, Tr, Ju, Cr, Pa, and N indicate the Devonian, Carboniferous, Permian, Triassic, Jurassic, Cretaceous, Paleogene, and Neogene, respectively. (E) Summary of rapidly evolving gene superfamilies. Green and pink numbers on each branch represent the number of significantly expanded and contracted gene families, respectively. The ancestor of the three leafhopper species is indicated by a red star. (F) Heatmap showing the number of genes in each superfamily that are expanded in the focal branch.

Using a species phylogeny and calibrated divergence times (approximately 174.72 Mya for Cicadellidae and 12.84 Mya for *Nephotettix*), we assessed the expansion and contraction of superfamilies in Hemiptera. Considering that these three species are widespread pests and can carry and transmit rice viruses and other pathogens [7, 8], we focused on superfamilies that underwent rapid evolution in the ancestors of the three species, including eight significantly expanded superfamilies and six significantly contracted superfamilies (Figure 1D,E). Among the expanded gene families, we found that the genomes of the three leafhopper species contained the thiol‐activated cytolysin superfamily (CDC, IPR036359), members of which encode a toxin produced by gram‐positive bacteria that has not been found in other insect genomes [20, 21, 22] (Figure 1F). Given that *CDC* genes have thus far been identified only in bacterial lineages, we hypothesized that leafhopper *CDC* genes may have been acquired from bacterial donors through horizontal gene transfer (HGT) and further tested this hypothesis using phylogenetic analysis.

### HGT of *CDC* genes from bacteria to leafhoppers

Genome annotation revealed that all three species had multiple *CDC* genes. In *R. dorsalis*, three *CDC* genes (*RdCDC6*, *RdCDC8*, and *RdCDC9*) had introns in their coding DNA sequences (CDSs), whereas at least two *CDC* genes (*RdCDC1* and *RdCDC7*) had introns in their 5'‐untranslated regions (5'‐UTRs). In contrast, all *CDC* genes in *Nephotettix* had only one exon in their CDSs, but transcriptome data showed that all of these genes had one intron in their UTRs (Figures 2A,B, S2, and S3). To confirm the existence of *CDC* genes in the genomes of these leafhoppers, we cloned all the *CDC* genes from genomic DNA and quantified their expression levels via polymerase chain reaction (PCR) and reverse transcription‐quantitative polymerase chain reaction (RT‐qPCR) (Figure 2C). We also performed immunoprecipitation (IP) of the candidate proteins from the lysate of *R. dorsalis* using IgG and RdCDC9 antibodies. The IP products were separated via sodium dodecyl sulfate‐polyacrylamide gel electrophoresis (SDS‐PAGE) and analyzed by liquid chromatography‐tandem mass spectrometry (LC‐MS/MS), and the RdCDC9 fragments were identified using an anti‐RdCDC9 antibody but not IgG (Figure 2D).

**FIGURE 2:**
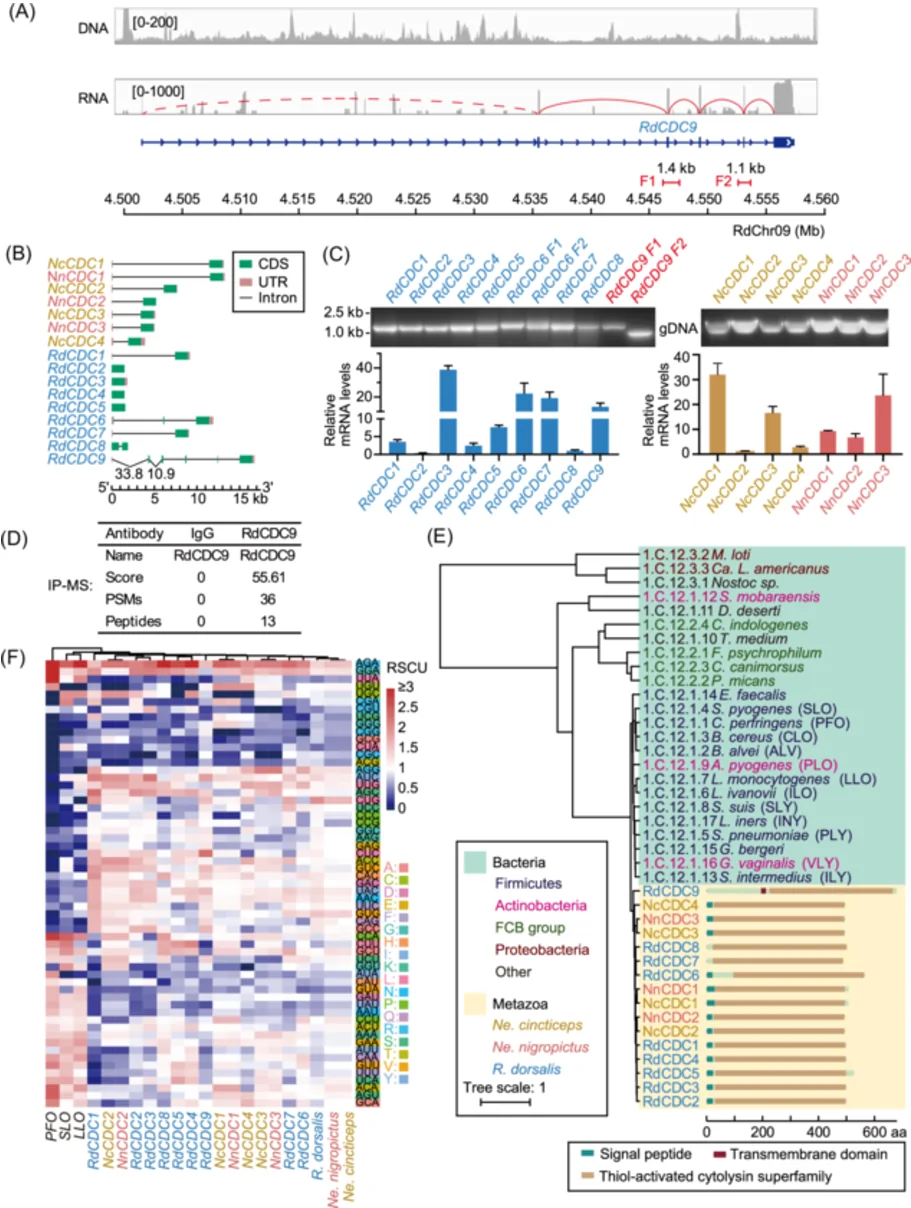
Signatures of genes suspected to be horizontally transferred from bacteria to leafhoppers. (A) Genome and transcriptome coverage of *RdCDC9* (*Id09G000860*). The read depths of genome resequencing and RNA sequencing data are log‐transformed and plotted along the focal genomic region, with numbers indicating the range of depths. Splice junctions in *RdCDC9* inferred from the RNA sequencing reads are indicated by red lines. The two fragments used for PCR cloning of genomic DNA are represented as red segments. (B) Gene structures of the *CDC* genes in three leafhopper species. Green and pink rectangles represent the CDSs and UTRs, respectively, and the black lines represent introns. (C) Genome fragments cloned from *Ne. cincticeps*, *Ne. nigropictus*, and *R. dorsalis* genomic DNA. For the intron‐bearing genes *RdCDC6* and *RdCDC9*, two pairs of primers targeting the intron regions were used. Bar plots represent the expression levels of all *CDCs* detected by RT‐qPCR. (D) Identification of the RdCDC protein by immunoprecipitation‐mass spectrometry (IP‐MS). The *R. dorsalis* adult lysate was immunoprecipitated using IgG or an RdCDC9 antibody and analyzed by mass spectrometry to identify candidate proteins. (E) Hierarchical clustering results of *CDC* family members based on protein similarity. All genes from leafhoppers are clustered together and grouped with the *CDCs* from Firmicutes and Actinobacteria, suggesting that they may have originated from an ancient horizontal gene transfer event. The TCDB accession number and the common name or species of origin are provided for each protein used to construct the phylogeny. Predictions of the protein domains revealed that all leafhopper *CDCs* contained a region belonging to the thiol‐activated cytolysin superfamily (IPR036359). (F) Heatmap showing the average relative synonymous codon usage (RSCU) of leafhopper *CDCs*, bacterial *CDCs* (*PFO*, *LLO*, and *SLO*), and conserved single‐copy genes of the three leafhopper species. The gradient from dark blue to dark red indicates increasing RSCU values from low to high. LLO, listeriolysin O; PFO, perfringolysin O; SLO, streptolysin O.

The 16 CDC proteins ranged in length from 490 to 680 amino acids (aa), 13 of which were predicted to be secreted proteins because they had signal peptides at their N‐termini (Figure 2E). The C‐terminus was identified as the thiol‐activated cytolysin domain, which is found only in bacteria [21]. To determine the origin of the leafhopper *CDC* genes, we constructed a phylogenetic tree based on sequences from the related membrane attack complex/perforin (MACPF)/CDC superfamily [21, 22]. Consistent with previous results, proteins in the *CDC* family (Transporter Classification Database: TCDB ID 1.C.12), *MACPF* family (TCDB ID 1.C.39), and *pleurotolysin B* family (TCDB ID 1.C.97.1) clustered separately. Moreover, phylogenetic analysis revealed that leafhopper *CDCs* were nested within the clade of classical *CDCs* from gram‐positive bacteria, including Firmicutes and Actinobacteria (TCDB ID 1.C.12.1). In contrast, *CDC* homologs from the FCB group and Proteobacteria (TCDB ID 1.C.12.2 and TCDB ID 1.C.12.3) formed the basal lineages (Figures 2E and S4). The placement of leafhopper *CDCs* within a bacterial lineage indicates that these genes were derived from a bacterial ancestor. This nested phylogenetic pattern strongly supports a bacteria‐to‐insect HGT event.

Additionally, relative synonymous codon usage (RSCU) values showed that the codon usage bias pattern of leafhopper *CDCs* significantly differed from that of bacterial *CDCs* (perfringolysin [*PFO*], listeriolysin [*LLO*], and streptolysin [*SLO*]) but was similar to that of other conserved single‐copy genes in the three leafhopper species (Figure 2F), indicating that the codon usage of the transferred genes has been domesticated and evolved to be consistent with that of the recipient genome.

To further investigate the evolutionary history of the insect *CDCs*, we first searched all available insect genomes in the NCBI RefSeq database for *CDC* homologs. Among the 322 insect species examined, *CDC* homologs were identified only in one additional leafhopper species, *Macrosteles quadrilineatus* (Table S3). Because *CDC* genes were also found in leafhopper species beyond the three species included in our initial analysis, we expanded our screening to all available genome resources from the family Cicadellidae to characterize the distribution of *CDC* genes within leafhoppers. Among the 29 leafhopper species analyzed, *CDC* homologs were detected in all 16 sampled species of the subfamily Deltocephalinae, whereas no homologs were identified in sampled species from other subfamilies, suggesting that the gene transfer event likely occurred in the common ancestor of Deltocephalinae (Figure S5A; Table S4). The copy number of *CDC*s varied between 3 and 14, and most gene copies were lineage‐specific, which indicates *CDC* genes are highly divergent and rapidly evolved after HGT (Figure S5B).

To further investigate the characteristics of these leafhopper *CDCs*, we examined the signatures of selection. Pairwise Ka/Ks ratios showed that some *CDCs*, especially *RdCDC9*, had higher nonsynonymous substitution rates, indicating that they may have undergone relaxed negative selection or positive selection (Figure S6A). We also evaluated selection pressure using the PAML branch‐site model and found that five leaf nodes in *R. dorsalis* (*RdCDC4*, *RdCDC5*, *RdCDC6*, *RdCDC8*, and *RdCDC9*) and internal nodes in *Nephotettix* showed signatures of positive selection (*p* < 0.05, Figure S6B), indicating the leafhopper *CDCs* may have undergone rapid adaptive evolution in the *Nephotettix* ancestor and *R. dorsalis*. Interestingly, the putatively selected site in RdCDC9 (C586, Bayes empirical Bayes analysis, *p* < 0.05) was a key amino acid in loop L2. L2 was one of four conserved motifs (loops L1 to L3 and the undecapeptide) responsible for cholesterol recognition and binding in bacterial CDCs [21]. Therefore, we checked the sequence alignments of the four motifs and found that the sequences were conserved in most leafhopper CDCs (Figure S7A), suggesting that these leafhopper CDC proteins have functions similar to those in bacteria. However, some gene copies have mutations in the conserved functional sites. More specifically, the cholesterol recognition motif (CRM, Thr‐Leu pair) in loop L1 was mutated in RdCDC7 and RdCDC9, and three proteins (RdCDC7, RdCDC9, and NcCDC4) had insertions or deletions in the undecapeptide region (Figure S7A). Additionally, RdCDC7, RdCDC8, and RdCDC9 lacked a signal peptide, and RdCDC9 had a potential transmembrane domain (Figure 2E). We also predicted these protein structures using AlphaFold 2 [23] and found that some RdCDCs, such as RdCDC6 and RdCDC9, exhibited obvious variations (Figure S7B). In summary, our results indicate that leafhopper *CDCs* have been domesticated and undergone sequence and functional differentiation after HGT from bacterial donors. Specifically, some *CDCs*, including *NcCDC4*, *RdCDC7*, and *RdCDC9*, may have been extensively domesticated in leafhoppers and may have partially or entirely lost their cholesterol‐binding and membrane‐anchoring ability and gained new functions. Conversely, other *CDCs*, such as *RdCDC3*, *NcCDC1*, and *NnCDC3*, did not exhibit signatures of relaxed negative selection or positive selection and may have maintained their original functions.

### *RdCDCs* exhibit *ex vivo* cytotoxic differentiation

We next wondered whether the conserved *CDCs* retained functions similar to those of the bacterial *CDCs*. We observed that *RdCDCs* were dispersed throughout the genome. For instance, *RdCDC3* and *RdCDC9* are located on chromosome 9, which is the sex chromosome, whereas *RdCDC6* is located on autosomal chromosome 8 (Figure 3A). We selected three representative *CDCs*, *RdCDC3*, *RdCDC6*, and *RdCDC9*, for functional research because they belong to three different *CDC* clusters that exhibit clear sequence and structural differentiation (Figures 3B, S7A, and S7B).

**FIGURE 3:**
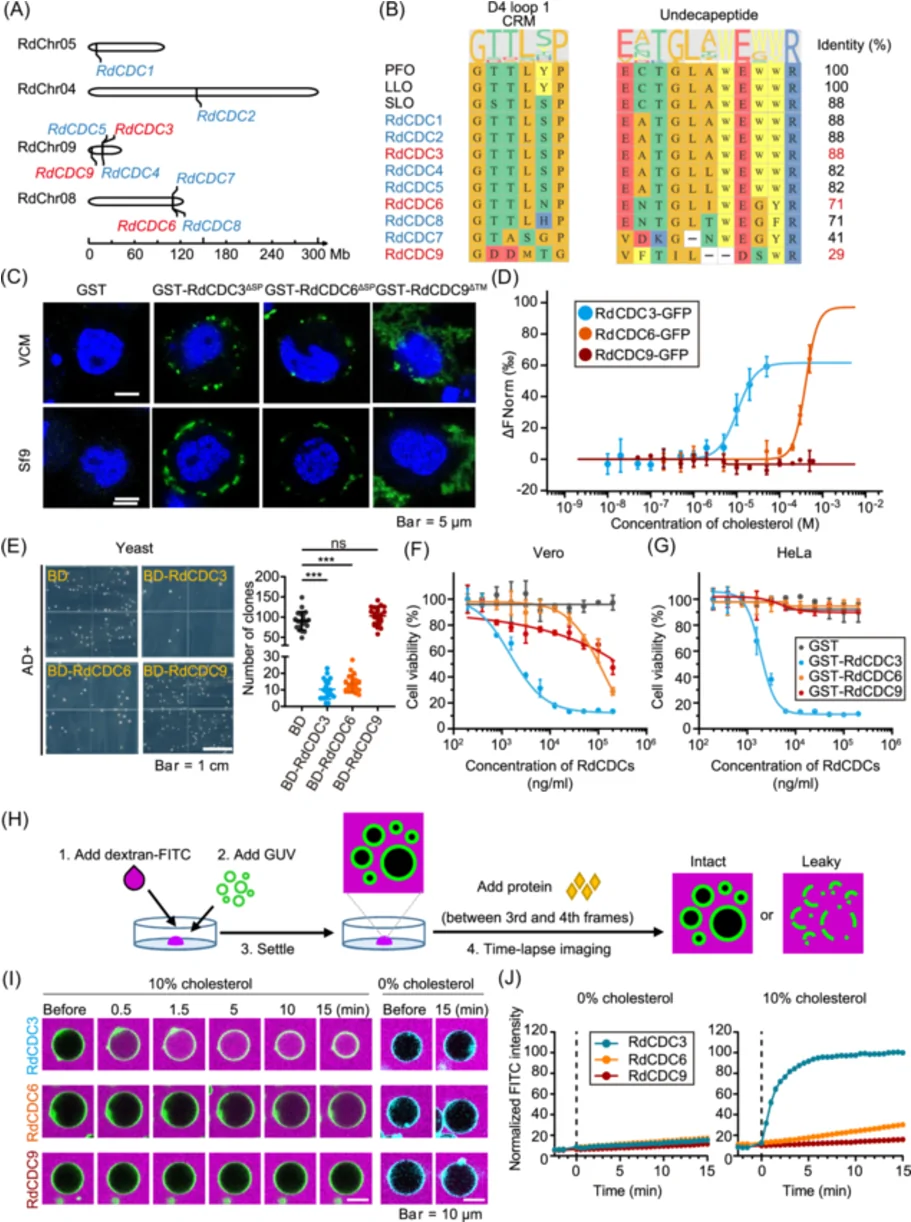
Cytotoxicity and cholesterol binding activity of *RdCDCs*. (A) Localization of the *RdCDCs* on chromosomes. *RdCDC3* and *RdCDC9* are located on chromosome 9, which is the sex chromosome, and *RdCDC6* is located on chromosome 8. (B) Amino acid sequence alignments of leafhopper and bacterial CDCs in the Loop 1 and undecapeptide regions. The amino acid sequence identities (%) of Loop 1 and the undecapeptide regions of SLO, LLO, and RdCDC were calculated with PFO as the reference. Background colors represent amino acid properties. (C) Immunofluorescence staining shows that the RdCDC3 and RdCDC6 proteins, lacking a signal peptide, bind to the insect cell membrane, whereas the RdCDC9 protein, which lacks a transmembrane domain, spreads throughout the pericellular space. GST‐RdCDCs were detected using an anti‐GST antibody. Nuclei were labeled with DAPI. Bar = 5 µm. (D) Assessment of cholesterol binding in the RdCDCs. Microscale thermophoresis demonstrated direct interactions between GFP‐tagged RdCDCs and cholesterol (after 15 min of incubation) in lysates from RdCDC‐GFP‐expressing Sf9 cells. Blue and orange fitting curves represent the cholesterol‐binding abilities of RdCDC3 and RdCDC6, respectively. The red line shows that RdCDC9 cannot bind cholesterol. ΔFNorm, normalized fluorescence. MST laser power: 20%; LED power: 40%. (E) Toxicity of the RdCDCs to yeast cells. AD plasmids with BD‐RdCDC3, BD‐RdCDC6, and BD‐RdCDC9 were cotransformed into the AH109 strain, which was subsequently coated on plates deficient in leucine and tryptophan. The colonies were then counted. Bar = 1 cm. ***p < 0.001. (F, G) The sensitivities of Vero and HeLa mammalian cells to RdCDCs were analyzed using MTT assays. The cells were plated in 96‐well microplates and exposed to RdCDCs for 24 h. The absorbance of the generated formazan was measured at 493 nm using a microplate reader. Cell viability curves were analyzed using Origin software (OriginPro 2020). Data are presented as the mean ± SEM from three independent biological replicates. (H) Schematic illustration of the GUV perforation assay. A drop of FITC‐dextran was added to a glass‐bottom confocal chamber. Another drop of GUVs was added and mixed gently with dextran. Droplets were allowed to settle at room temperature for 0.5–1 h to ensure maximal attachment of GUVs to the glass surface. Time‐lapse imaging was performed, with protein added during a 60‐s pause between the 3rd and 4th frames. A 15‐min movie was then recorded to capture the dynamics of perforation. GUV perforation was characterized as an elevation of the FITC signal inside GUVs. (I) Representative time‐lapse confocal images showing GUVs containing 10% (left panel) or 0% (right panel) cholesterol treated with 400 nM RdCDC3 (top panel), RdCDC6 (middle panel), or RdCDC9 (bottom panel). Time points are indicated at the top of the corresponding images. See also Video S1. Magenta represents FITC‐dextran, and green or cyan represents rhodamine‐PE incorporated into GUVs containing 10% or 0% cholesterol, respectively. Scale bars, 10 μm. (J) Statistical analysis of GUV perforation in (I). The mean FITC‐dextran intensities inside GUVs over time were normalized to the FITC intensity in solution and plotted as a function of time. The dashed line indicates the time point of protein addition. Data are presented as the mean ± SEM. *N* = 116, 111, and 113 GUVs were analyzed for 10% cholesterol GUV treated with RdCDC3, RdCDC6, or RdCDC9, respectively. *N* = 56, 56, and 59 GUVs were analyzed for 0% cholesterol GUVs treated with RdCDC3, RdCDC6, or RdCDC9, respectively.

To investigate the physicochemical functions of these three *RdCDCs*, we initially transfected *Spodoptera frugiperda* 9 (Sf9) cells with recombinant baculoviruses [24]. Subsequently, we examined the expression and localization of these proteins using laser confocal microscopy. We observed that RdCDC3, RdCDC6, and RdCDC9 all formed granular aggregates in the plasma membrane (Figure S8A). Given that cholesterol is anchored within the cell membrane, we investigated the binding of RdCDCs to the cell membrane. We generated the recombinant proteins RdCDC3^*ΔSP*^, RdCDC6^*ΔSP*^, and RdCDC9^*ΔTM*^ tagged with glutathione S‐transferase (GST) at their N‐termini and cultured the proteins with Sf9 cells or vector cell monolayers (VCMs) from *Ne. cincticeps*. Through immunostaining with a GST antibody and subsequent fluorescence detection, we observed that GST‐RdCDC3 and GST‐RdCDC6 bound to the plasma membrane, whereas GST‐RdCDC9 did not (Figure 3C). Next, we used microscale thermophoresis (MST) experiments to measure the binding activity of the three RdCDCs to cholesterol, and the results revealed that RdCDC3‐GFP directly interacted with cholesterol in Sf9 cell lysates with an affinity (Kd) of 6.83 × 10^−6^ M. However, RdCDC6 exhibited weak binding activity, and RdCDC9 completely lost binding activity (Figures 3D and S8B).

To further evaluate the cytotoxicity of these RdCDCs, we first performed experiments in yeast cells. We cotransformed and expressed activation domain (AD)/binding domain (BD)‐RdCDC3, AD/BD‐RdCDC6, and AD/BD‐RdCDC9 in the AH109 strain. We found that, compared with control yeast, the yeast cotransformed with AD/BD‐RdCDC9 grew normally, whereas the growth of the yeast cotransformed with AD/BD‐RdCDC6 was strongly suppressed, and there was almost no growth of the yeast cotransformed with AD/BD‐RdCDC3; moreover, we quantified these observations by counting colonies (Figure 3E). To validate the cytotoxicity of these *RdCDCs*, we microinjected these recombinant RdCDC proteins into fourth‐instar larvae of *Bombyx mori*. Similarly, RdCDC3 significantly reduced the survival of *B. mori* in a dose‐dependent manner, whereas RdCDC6 and RdCDC9 displayed effects only at extremely high doses (Figure S8C). To confirm these findings, we conducted 3‐(4,5‐dimethylthiazol‐2‐yl)−2,5‐diphenyltetrazolium bromide (MTT) assays [20] to measure the viability of cells from various cell lines, including HeLa, Vero, HEK293T, and Sf9 cells. We cultured the cells in the presence of the recombinant fusion proteins GST‐RdCDC3, GST‐RdCDC6, and GST‐RdCDC9 purified from *Escherichia coli* for 24 h at the appropriate culture temperature. Surprisingly, only RdCDC3 exhibited potent toxicity to Vero, HeLa, and HEK293T cells, with doses leading to 50% cell death (IC_50_) of approximately 1–3 µg/mL; these proteins had relatively weak toxicity to Sf9 cells (Figures 3F,G, and S8D–S8G). Overall, RdCDC3 displayed a strong ability to bind cholesterol, caused a high mortality rate in *B. mori*, and was highly toxic to Vero, HeLa, and HEK293T cells. In contrast, RdCDC6 and RdCDC9 bound cholesterol weakly or not at all, were nontoxic to Vero and HeLa cells, and reduced the survival rate of *B. mori* at extremely high dosages.

To further investigate the role of cholesterol in RdCDC perforation, we established a giant unilamellar vesicle (GUV)‐based in vitro reconstitution system. The simplicity of in vitro‐synthesized GUVs allowed us to create cell‐mimicking vesicles with precisely controlled cholesterol concentrations while avoiding interference from other components in cells (Figure 3H).

We prepared rhodamine‐phosphatidylethanolamine (PE)‐labeled GUVs containing 0% to 20% cholesterol and used membrane‐impermeable fluorescein isothiocyanate (FITC)‐dextran as an indicator of GUV leakage. We found that the addition of RdCDC3 had distinct effects on GUVs with different cholesterol concentrations. Although GUVs without cholesterol showed no obvious dextran penetration or morphological changes after RdCDC3 treatment, GUVs containing 10% or 20% cholesterol showed immediate penetration of dextran. Moreover, with 10% cholesterol, gradual deformation was observed after penetration. With 20% cholesterol, simultaneous rupture was observed in 48% of GUVs. These results clearly revealed that cholesterol is essential for RdCDC3 perforation and that the kinetics are positively correlated with the cholesterol concentration on the target membrane. For the other two proteins, RdCDC6 showed a cholesterol‐dependent perforation effect with a much lower efficiency relative to that of RdCDC3, whereas RdCDC9 showed no significant perforation effect under any conditions (Figures 3I,J, S9A–S9F, and Videos S1–S2). To further compare perforation efficiency between bacterial CDCs and leafhopper CDCs, we selected *pyolysin* (PLO), which belongs to the bacterial CDCs and does not require thiol activation, similar to RdCDC3. Using the GUV‐based in vitro reconstitution system, we found that, at the same cholesterol concentration, the perforation effect of RdCDC3 was stronger and faster than that of PLO (Figure S9G, S9H, and Video S3), suggesting that RdCDC3 had evolved higher cytotoxicity than the original bacterial CDCs.

### RdCDC3 is specifically expressed in the serosa of eggs and exhibits inhibitory effects on the feeding of natural leafhopper predators

As presented in the above section, our study provides strong evidence supporting the hypothesis that HGT events provided leafhopper species with CDCs exhibiting variable cytolytic activity, which prompted us to further decipher the ecological roles of these leafhopper *CDCs*. First, we examined the expression of these *CDCs* in leafhoppers of different sexes, and surprisingly, we found that *RdCDC3* was predominantly expressed in females (Figure 4A). Further specific organ detection via reverse transcription‐polymerase chain reaction (RT‐PCR) and RT‐qPCR revealed that *RdCDC3* mRNA was highly expressed in the ovaries and early‐stage eggs, whereas *RdCDC6* and *RdCDC9* were mainly expressed in the gut (Figure 4B,C). Interestingly, western blot analysis using an anti‐RdCDC3 antibody showed that the RdCDC3 protein was consistently expressed throughout the egg stage, with actin serving as an internal reference (Figure 4C). To further determine the localization of RdCDC3, we used western blotting, immunofluorescence, and immunogold labeling of ultrathin sections of eggs with an anti‐RdCDC3 antibody. The immunofluorescence and immunogold labeling results were obtained via laser confocal microscopy and transmission electron microscopy (TEM), respectively. To aid in understanding these results, we provided a picture of an egg, a schematic diagram of an egg, and an ultrathin section of the eggshell and serosa cuticle that illustrate the organization of the egg in Figure 4D. The immunofluorescence and western blot results showed that RdCDC3 was specifically expressed in the eggshell but not in the egg content (EC) (Figure 4E,F), and the immunogold labeling results further demonstrated that RdCDC3 was specifically expressed in the serosa (S) (Figure 4G).

**FIGURE 4:**
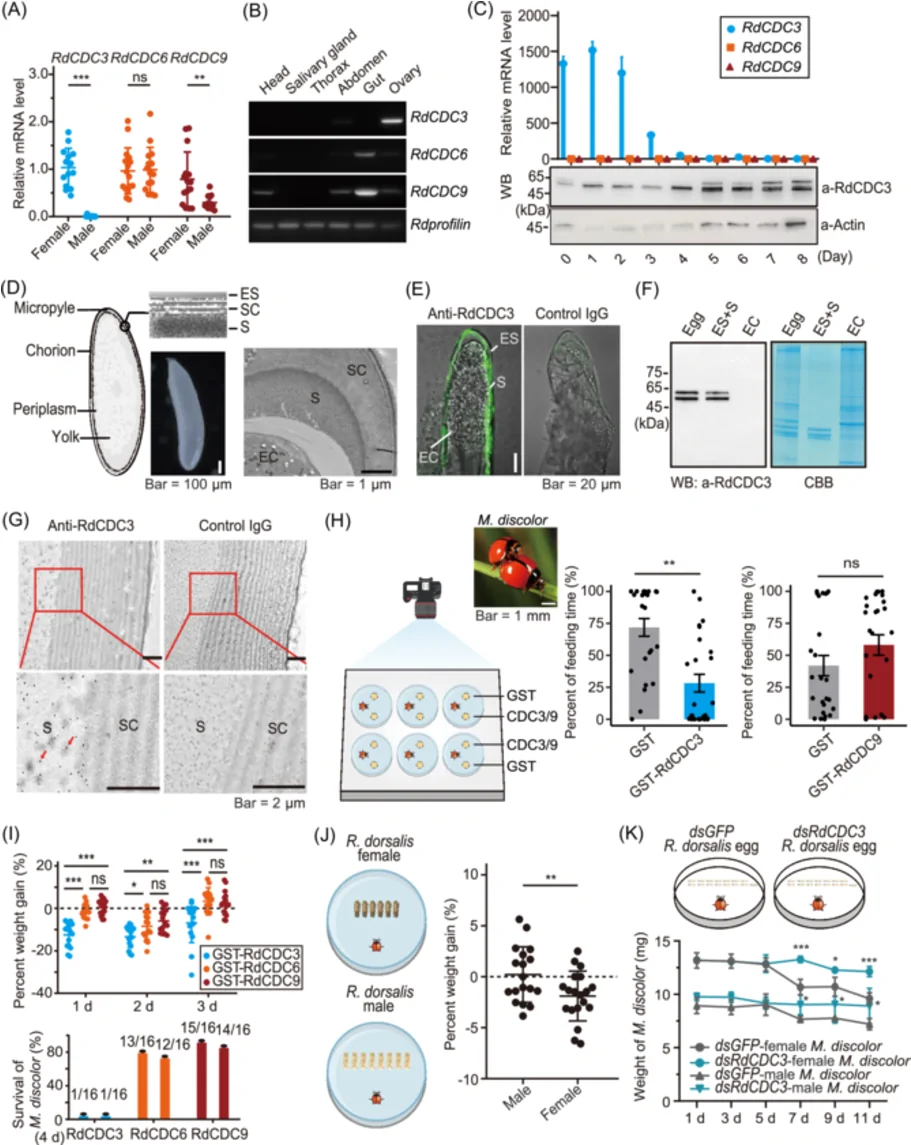
*RdCDC* expression patterns and the effects of *RdCDC3* on ladybug feeding. (A) Expression patterns of *RdCDC3*, *RdCDC6*, and *RdCDC9* in male and female *R. dorsalis* detected via RT‐qPCR. **p < 0.01; ***p < 0.001. *N* = 16. (B) Tissue‐specific expression patterns of *RdCDC3*, *RdCDC6*, and *RdCDC9* detected by semiquantitative RT‐PCR after 30 cycles. Twenty female *R. dorsalis* individuals were dissected to separate different tissues, including the head, salivary gland, thorax, abdomen, gut, and ovary. (C) Temporal expression profiles and spatiotemporal protein accumulation data. The expression patterns of *RdCDC3*, *RdCDC6*, and *RdCDC9* in eggs at different stages were detected using RT‐qPCR, and accumulation of the RdCDC3 protein in eggs at different stages was detected via western blotting using an antibody against RdCDC3. (D) Schematic of the egg profile showing the pattern organization of the egg at four to five days postoviposition. The main tissues included the micropyle, chorion, periplasm, and yolk. The peripheral embryonic organization, including the serosa, serosal cuticle, and eggshell, typically plays a defensive role. A photograph of an *R. dorsalis* egg (bar = 100 µm) and an ultrathin section of the egg depicting the pattern organization (bar = 1 µm) are shown. ES: eggshell; SC: serosal cuticle; S: serosa; EC: egg content. (E) Immunofluorescence detection of the location of the RdCDC3 protein in the organizational structure of the eggs using an antibody against RdCDC3; IgG served as a control. Images were taken with a ZEN laser confocal microscope, and GFP was visualized at an excitation wavelength of 488 nm. Bar = 20 µm. (F) Detection of the accumulation of RdCDC3 protein in extraembryonic tissue, including the eggshell and egg contents, via the use of an antibody against RdCDC3. Coomassie Brilliant Blue (CBB) staining of total protein loading in the egg samples. (G) Immunogold labeling was used to detect the location of RdCDC3 in the outer layer of the eggs with an antibody against RdCDC3; IgG was used as a control. The diameter of the colloidal gold particle was 10 nm. The labeled ultrathin eggshell sections were imaged via transmission electron microscopy. Bar = 2 µm. (H) Feeding test of *M. discolor* with food containing different CDC proteins. *Ephestia kuehniella* eggs and *Brassica campestris* pollen mixed with GST‐RdCDC3/GST‐RdCDC9 or GST proteins were placed on each side of a Petri dish. A video recorder was used to record the behavior of the *M. discolor* individual, and feeding time was calculated using the OpenCV module in Python. The device for observing the feeding preferences of *M. discolor* is shown on the left*. N* = 25. The statistical significance of the fixed effects was assessed by ANOVA of LMMs. **p < 0.01. (I) Effects of CDC protein injection on the growth and survival of *M. discolor*. Individuals were microinjected with RdCDC3, RdCDC6, or RdCDC9, and percent weight gain was determined at 1, 2, and 3 days after injection (top panel; *N* = 16). Statistical significance was assessed using an LMM, followed by post hoc pairwise comparisons among groups at each time point with Tukey adjustment for multiple comparisons. *, adjusted *p* < 0.05; **, adjusted *p* < 0.01; ***, adjusted *p* < 0.001. Survival rates were determined at 4 days after injection (bottom panel). (J) Feeding assay of *M. discolor* with male or female leafhoppers. Six female or eight male leafhoppers were placed at the bottom of a plastic dish with one *M. discolor* individual, and changes in *M. discolor* body weight were measured. *N* = 18 and 19 for males and females, respectively. **p < 0.01 (Student's *t* test). (K) Effects of feeding on eggs from *RdCDC3* knockdown leafhoppers on the body weight of *M. discolor*. Eggs were collected from pregnant female leafhoppers treated with *dsGFP* or *dsRdCDC3* and provided to *M. discolor* every 2 days. Body weights of *M. discolor* were measured on Days 1, 3, 5, 7, 9, and 11. Statistical significance was assessed using an LMM, followed by post hoc pairwise comparisons among groups at each time point for each sex of *M. discolor*, with Tukey adjustment for multiple comparisons. *, adjusted *p* < 0.05; ***, adjusted *p* < 0.001.

The specific developmental stage and serosa expression of *RdCDC3* suggest that expression of the toxin gene is under precise regulation, which, on one hand, avoids being toxic and harmful to the leafhopper itself. On the other hand, such regulation may be useful for defending the eggs and offspring, which are essential for maintaining leafhopper populations, against natural enemies. To verify this hypothesis, we evaluated the effect of *RdCDC3* on *Micraspis discolor* (ladybugs), a natural predator that mainly feeds on leafhoppers in rice fields, using in vitro and in vivo experiments. We first examined whether the RdCDC3 protein affected the feeding preferences of the ladybugs. We mixed GST‐RdCDC proteins with the artificial food of *M. discolor*, which consisted of *Ephestia kuehniella* eggs and *Brassica campestris* pollen [25], with the GST protein serving as a control. We recorded the feeding time of *M. discolor* using a real‐time monitoring camera to evaluate feeding preference (Figure 4H, left). A linear mixed‐effects model (LMM) revealed a significant effect of food type on feeding time (*F* = 7.63, *p* = 0.008), whereas neither food position nor the interaction between food type and position had a significant effect (*F* = 0.01, *p* = 0.92; *F* = 0.22, *p* = 0.64, respectively). After accounting for the effect of position, feeding time was significantly shorter for food containing GST‐RdCDC3 than for the GST‐only control (Figure 4H, middle). In contrast, GST‐RdCDC9 showed a marginal effect on feeding time (*F* = 4.02, *p* = 0.05), with a trend toward increased feeding time relative to the GST‐only control (Figure 4H, right), suggesting that the predator exhibited a negative preference for the RdCDC3 protein. Therefore, we subsequently investigated whether the RdCDC3 protein would have a detrimental effect on the predator by microinjecting the GST‐RdCDC proteins into *M. discolor*. Both group and time significantly affected percent weight gain (LMM, group: *F* = 41.32, *p* < 0.001; time: *F* = 28.01, *p* < 0.001), whereas no significant group × time interaction was detected (*F* = 1.70, *p* = 0.15). Consistent with these overall effects, post hoc pairwise comparisons showed that *M. discolor* individuals injected with RdCDC3 exhibited significantly lower percent weight gain than those injected with RdCDC6 or RdCDC9 at all three time points (all adjusted *p* < 0.05) (Figure 4I, top). At 4 days post‐injection, almost all *M. discolor* individuals that had been injected with RdCDC3 had died, whereas the majority of those injected with RdCDC6 and RdCDC9 were still alive (Figure 4I, bottom), indicating that RdCDC3 was toxic to the ladybugs. To further confirm whether RdCDC3 proteins at physiological concentrations in leafhoppers had effects similar to those of purified proteins, we conducted a feeding trial in *M. discolor* by providing either pregnant female *R. dorsalis* or males that expressed or did not express *RdCDC3*, respectively (Figure 4J, left). The results showed that the percent weight gain of *M. discolor* preying on female leafhoppers was significantly lower than that of *M. discolor* preying on male leafhoppers (Figure 4J, right, *p* < 0.01, Student's *t* test), indicating that feeding on female leafhoppers was harmful to the growth of *M. discolor* and thus that female leafhoppers might not be preferred by predators under natural conditions. To rule out other possibilities resulting in differences between male and female leafhoppers, we next directly validated the protective function of *RdCDC3* on eggs by inhibiting gene expression in vivo. We fed *M. discolor* with leafhopper eggs from the *RdCDC3* knockdown individuals and control groups and recorded changes in weight over time. RT‐qPCR analysis confirmed efficient silencing of *RdCDC3* in eggs from the knockdown group (Figure S10A and S10C). The results showed that group, time, and sex of *M. discolor* all significantly affected body weight (LMM, group: *F* = 11.56, *p* = 0.009; time: *F* = 20.16, *p* < 0.001; sex: *F* = 118.68, *p* < 0.001). A significant group × time interaction was also detected (*F* = 6.91, *p* < 0.001), indicating that the effect of group on body weight varied over time. Post hoc pairwise comparisons further showed that, during the later stages of the experiment (Days 7, 9, and 11), both male and female *M. discolor* feeding on eggs that lacked *RdCDC3* had significantly higher body weights than their respective controls (all adjusted *p* < 0.05) (Figure 4K), supporting the conclusion that the presence of *RdCDC3* effectively reduced the predation or utilization of leafhopper eggs by ladybugs. In summary, the above results suggest that the RdCDC3 protein is an important factor in making female leafhoppers and eggs relatively unpalatable and toxic to predators, demonstrating its crucial role in protecting leafhoppers from consumption.

### RdCDC9 is specifically expressed in the gut of leafhoppers and maintains homeostasis of the gut microbiome

The above results demonstrated that RdCDC3 retained the characteristics of bacterial *CDCs*, such as protein structure and toxic function. However, *RdCDC9* appears to have been domesticated and evolved more like a leafhopper gene, containing introns and losing cholesterol‐binding activity as well as cytotoxicity, suggesting that it may have evolved new functions. Therefore, we further explored the functions of *RdCDC9* in leafhoppers. First, we examined the expression pattern of *RdCDC9* and found that it was highly expressed in the gut (Figure 5A), which is a key organ in insect nutrient absorption, immune regulation, and microbial interactions. Thus, we evaluated whether *RdCDC9* influenced the growth, development, or reproduction of leafhoppers. We silenced *RdCDC9* by RNA interference (RNAi), and the expression of *RdCDC9* was significantly suppressed (Figure S10B and S10D). Surprisingly, we found that the knockdown of *RdCDC9* led to a significant reduction in weight gain (Student's *t* test, *p* < 0.001, Figure 5B). Survival was also significantly lower in the treatment group than in the control group (quasibinomial generalized linear model [GLM], *F* = 11.10, *p* = 0.03, Figure 5C). Additionally, treatment significantly affected offspring number (Poisson GLM, χ^2^ = 11.16, *p* < 0.001), with the mean number of offspring decreasing from 47.7 in the control group to 30.7 in the treatment group, corresponding to an approximately 35.7% reduction in offspring production (Figure 5D). Together, these results indicated that *RdCDC9*, despite its bacterial origin and lineage‐specific distribution, has acquired critical roles in multiple fitness‐related traits, including growth, survival, and reproduction.

**FIGURE 5:**
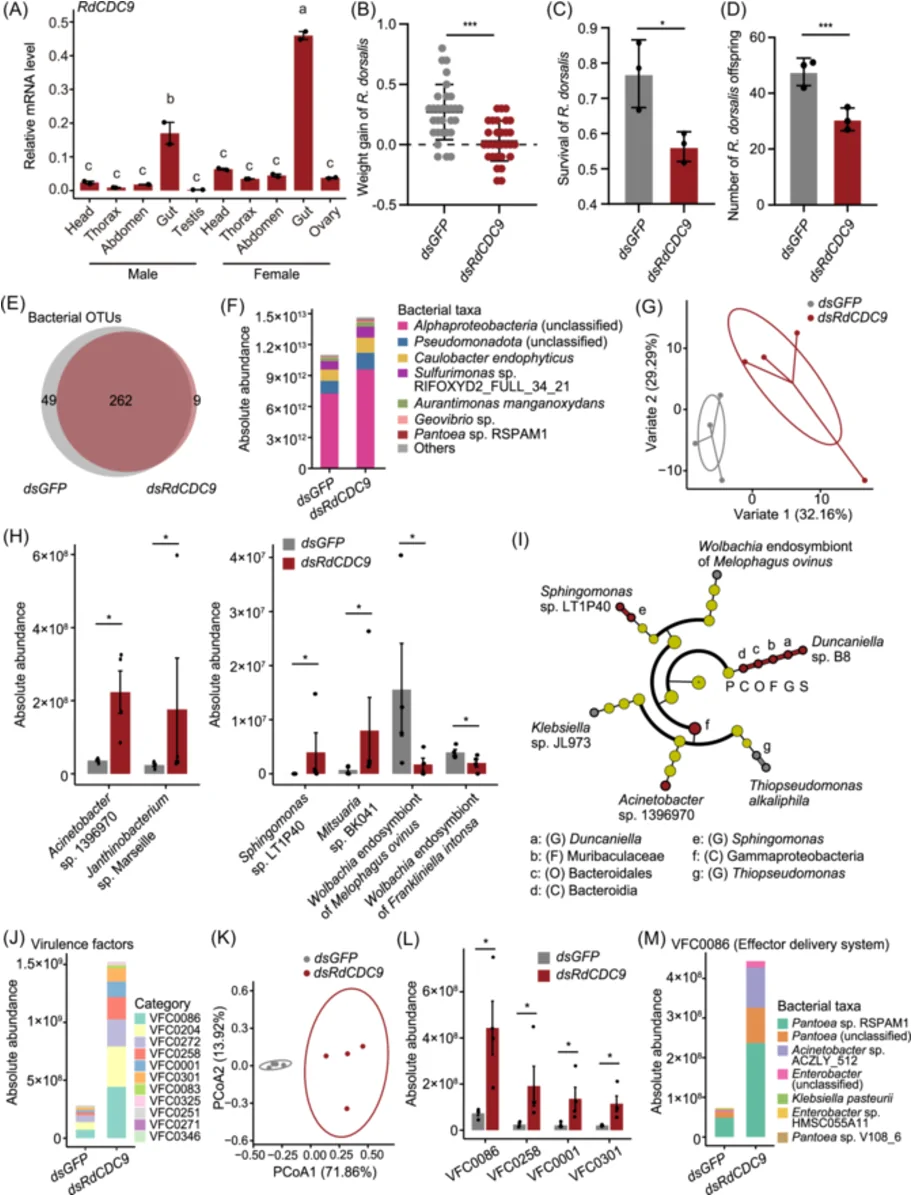
*RdCDC9* potentially maintains homeostasis of the gut microbiome and plays critical roles in insect survival. (A) The expression patterns of *RdCDC9* mRNA were quantified via RT‐qPCR in different tissues, including the head, thorax, abdomen, gut, testis, and ovary. Statistical significance was assessed using one‐way ANOVA followed by least significant difference (LSD) test for multiple comparisons. Different letters indicate significant differences between groups (*p* < 0.05). (B) Feeding assay of *dsGFP*‐ and *dsRdCDC9*‐microinjected leafhoppers. Weight gain was determined at 7 days after microinjection. *N* = 30 per group. ***p < 0.001 (Student's t test). (C) Survival rates of leafhoppers at 7 days after microinjection. Three biological replicates were performed, with 70 microinjected leafhoppers per replicate. Statistical significance was assessed using a quasibinomial GLM with an *F* test. *p < 0.05. (D) Numbers of offspring from *dsGFP*‐ and *dsRdCDC9*‐microinjected leafhoppers. At 2 days after microinjection, injected leafhoppers were transferred to fresh rice seedlings, and the number of offspring was counted after nymph hatching. Three biological replicates were performed. Each treatment group contained 10 males and 10 females. Statistical significance was assessed using a Poisson GLM with a likelihood‐ratio χ^2^ test. ***p < 0.001. (E) Numbers of bacterial OTUs detected in the gut of *dsRdCDC9*‐ and *dsGFP*‐microinjected leafhoppers. *N* = 4 per group. (F) Average absolute abundances of dominant bacterial OTUs at the species level. Only species with an average relative abundance greater than 0.5% are shown. (G) PLS‐DA score plot. The first two latent variables explained 32.16% and 29.29% of the variance, respectively. (H) Absolute abundances of bacterial OTUs with significant differences. Data are presented as the mean ± SEM. *p < 0.05 (Student's *t* test). (I) Cladogram showing the phylogenetic relationships of differentially abundant taxa between the two groups. Red and gray circles represent bacteria enriched in *dsRdCDC9* and *dsGFP* samples (LDA score > 2, *p* < 0.05), respectively. Concentric circles in the diagram, from inner to outer, sequentially represent taxa at the levels of phylum (P), class (C), order (O), family (F), genus (G), and species (S). Supraspecific taxonomic information is listed below the diagram. (J) Average absolute abundances of genes belonging to each category in the VFDB. *N* = 4 per group. (K) Principal coordinates analysis (PCoA) results based on the Bray‐Curtis distance matrix, which was calculated from the abundance profiles of VFDB categories. Circles represent 90% confidence intervals. PCoA1 and PCoA2 explained 71.86% and 13.92% of the variance, respectively. (L) Absolute abundances of VFDB categories with significant differences. Data are presented as the mean ± SEM. *p < 0.05 (Wilcoxon test). (M) Contributions of bacterial OTUs at the species level to effector delivery system (VFC0086)‐related genes.

Based on the gut‐specific expression patterns and important physiological functions of *RdCDC9*, we speculated that the silencing of *RdCDC9* may disrupt the gut microbial community of leafhoppers and thus affect their nutrient metabolism and survival. Therefore, we compared the gut microbiomes of *RdCDC9* knockdown (*dsRdCDC9*) and control (*dsGFP*) individuals using a quantitative metagenomic sequencing approach. After quality control, assembly, and redundancy removal, 1.15 × 10^6^ contigs with a total length of 883 Mb were obtained, and 4.5 × 10^5^ genes were annotated. On average, 44% of the sequences were assigned to bacteria (Figure S11A), whereas the relative abundances of all other microorganisms were less than 0.01%. Therefore, we focused on the gut bacterial community and detected 320 operational taxonomic units (OTUs), 262 of which were present in both *dsGFP* and *dsRdCDC9* samples (Figure 5E). Additionally, these samples showed no significant differences in community richness and diversity (Figure S11B), such that the top six most abundant bacterial OTUs were identical and an unclassified Alphaproteobacteria dominated the microbial community at approximately 66% relative abundance (Figure 5F). Principal coordinates analysis (PCoA) based on Bray–Curtis distance also showed that samples from the two groups partially overlapped (Figure S11C), and statistical testing via permutational multivariate analysis of variance (PERMANOVA) indicated no significant differentiation between groups (*F* = 0.24, *p* = 0.70). These results suggested the core microbiome was relatively stable in *RdCDC9* knockdown individuals. Nevertheless, we observed clear separation between the two groups by conducting supervised dimension reduction analysis, such as partial least‐squares discriminant analysis (PLS‐DA) (Figure 5G). Therefore, we wondered what bacteria drove the differences between groups and were associated with the silencing of *RdCDC9*. Student's *t*‐test showed that the absolute abundances of four OTUs were significantly increased in *dsRdCDC9* samples (*p* < 0.05), including members of the genera *Acinetobacter*, *Janthinobacterium*, *Sphingomonas*, and *Mitsuaria* (Figure 5H). In contrast, the abundances of two endosymbiont bacteria of the genus *Wolbachia* were decreased when *RdCDC9* was silenced (Figure 5H), which was also supported by linear discriminant analysis effect size (LEfSe) results (Figures 5I, S11D, and S11E). In addition to bacterial abundance profiling, we investigated the potential functions of the metagenome, with a particular focus on identifying factors that may be detrimental to leafhopper survival and reproduction. Thus, the functions of gene sets were annotated by searching the Virulence Factor Database (VFDB) (Figure S11F). Based on the abundance of genes related to virulence factors (Figure 5J), the samples were separated into two distinct clusters, which precisely corresponded to the *RdCDC9* knockdown and control groups, respectively (Figures 5K and S11G), and the observed difference was significant (PERMANOVA test, *F* = 12, *p* < 0.05; analysis of similarities [ANOSIM] test, *p* < 0.05). Specifically, the gene abundances of four VFDB categories, such as the effector delivery system (VFC0086), immune modulation (VFC0258), adherence (VFC0001), and regulation (VFC0301), were significantly higher in *RdCDC9* knockdown samples (Figure 5L, Wilcoxon test, *p* < 0.05). Among them, genes related to the effector delivery system had the highest abundance and mainly originated from the genera *Pantoea*, *Acinetobacter*, and *Enterobacter* (Figure 5M). Gene‐level analysis revealed six virulence factor genes with significantly increased abundance in *dsRdCDC9* samples, which were functionally categorized into effector secretion (including the type VI secretion system proteins *tssC* and *tssD*, as well as the outer membrane protein *ompA*) and adhesion and motility of pathogenic bacteria (the Tap pili component *tapT*, elongation factor *tufA*, and flagella‐related gene *cheY*) (Figure S11H). These results indicated that silencing *RdCDC9* altered the gut microbial community, leading to a reduction in beneficial endosymbiotic bacteria and the enrichment of potentially harmful bacteria carrying virulence factors. These microbial changes may partially contribute to the impaired growth and development observed in leafhoppers, suggesting that *RdCDC9* could potentially influence host fitness through modulation of the associated microbial community.

To summarize, the results from this research established the new concept that the domestication and diversification of horizontally transferred genes of bacterial origin empower leafhopper adaptation through diverse functions, including helping females avoid predators and ensure reproduction and maintaining gut microbiome homeostasis in a favorable state (Figure 6).

**FIGURE 6:**
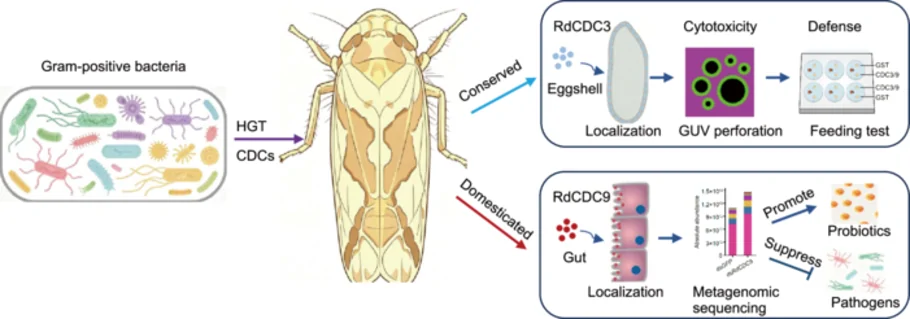
Schematic overview of the evolution and functional mechanism of leafhopper *CDCs*. *CDCs* originated from gram‐positive bacteria and were horizontally transferred into leafhoppers. These *CDCs* either maintained their original functions or were domesticated and obtained novel functions. *RdCDC3*, which retains cytolytic activity, is highly specifically expressed in the egg serosa and discourages predator feeding, thereby protecting offspring and perpetuating leafhopper populations. However, *RdCDC9* was domesticated and obtained novel function. Its cytotoxicity has decreased, and it is specifically expressed in the gut and regulates gut microbiota homeostasis to maintain leafhopper survival. The cyan dot indicated by the arrow represents the location of the RdCDC3 protein on the egg serosa; the red dot indicated by the arrow represents the location of the RdCDC9 protein in the intestinal epithelium; blue dots represent nuclei; and yellow dots represent probiotics, including *Wolbachia*.

## DISCUSSION

In this research, we constructed three high‐quality genome assemblies representing two genera in the largest subfamily of Cicadellidae, Deltocephalinae, which will facilitate research on the phylogenetic relationships, chromosome evolution, and genomic basis of adaptive traits in leafhoppers [26]. In the genomes of the three leafhopper species, we identified multiple genes encoding *CDCs*, which are of gram‐positive bacterial origin (Figures 1F and 2E). These *CDCs* exhibit high sequence and structural variation, as well as functional variation. Understanding the functions of insect genes acquired via HGT, especially in non‐model organisms, is challenging. Recently, a few case studies have shown that multiple independent HGT events have occurred in Hemiptera, and HGT events have been found to play critical ecological roles in insects by detoxifying toxins in food, overcoming plant defenses, and providing resistance to their parasitoid wasps [12]. HGT can also contribute to male courtship in lepidopterans [27].

Here, we present a unique HGT event that allowed us to perform a systematic study to trace the evolutionary history and characterize the functional features of homologous transferred genes in the same recipient genome [28, 29]. The results demonstrated that the functional diversification and domestication of these horizontally transferred genes have enabled leafhoppers to adapt to and combat their natural enemies and balance their life history.

Among these *CDCs*, *RdCDC3* exhibits typical bacterial *CDC* characteristics, such as strong cholesterol affinity and cytotoxicity, and the developmental stage and serosa expression specificity prevent it from damaging the leafhoppers themselves, instead making them unpalatable and toxic to the predator *M. discolor*. In contrast, *RdCDC9* is domesticated and more like a leafhopper gene; it no longer has cholesterol‐binding capacity or cytotoxicity, but it is specifically expressed in the gut of the leafhopper. Our analyses suggest that *RdCDC9* may contribute to maintaining gut microbial homeostasis and, consequently, influence leafhopper growth and development (Figure 6). We propose that *RdCDC9*‐mediated modulation of the gut microbiome represents a potential mechanism underlying these phenotypic effects. Nevertheless, the direct causal relationship between microbial alterations and host developmental outcomes remains to be established and requires further validation, for example, through experiments using gnotobiotic insect systems.

Additionally, *RdCDC6* is apparently in a transient state between *RdCDC3* and *RdCDC9* in terms of sequence, structure, and function. Thus, we propose that these horizontally transferred *CDCs* were domesticated and coevolved in leafhoppers through natural selection during long‐term interactions with other organisms in the environment. Pressures from domestication and natural selection may result in sequence, structural, expression pattern, and functional diversification. Therefore, by performing an in‐depth functional characterization of these horizontally transferred *CDCs*, our study systematically revealed that these genes have experienced varying degrees of domestication in the recipient genome, undergone functional differentiation, and jointly benefited the recipient species in multiple ways.

This study presents a groundbreaking exploration of HGT as a key driver of adaptation in insects, using leafhoppers (Cicadellidae) as a model system. The findings reveal a sophisticated evolutionary strategy in which leafhoppers have harnessed bacterial genes to enhance survival in their ecological niches. In general, the findings from this study may lead to an in‐depth understanding of the adaptation of the insect world to the environment and the development of novel strategies for the control of sap‐sucking pests and the diseases they transmit through targeting *CDC* genes and microbiome engineering.

## CONCLUSION

Leafhoppers acquired *CDC* genes from gram‐positive bacteria through HGT. These genes subsequently underwent functional diversification: conserved toxic *CDCs* are specifically expressed in the egg serosa and protect eggs against natural enemies, whereas nontoxic *CDCs* have evolved novel gut‐specific functions that likely contribute to microbiome homeostasis and promote leafhopper growth. Together, our findings demonstrate how the domestication and functional diversification of horizontally transferred genes can enhance host fitness through multiple mechanisms, highlighting HGT as an important source of evolutionary innovation in insects.

## METHODS

### Insect breeding

Leafhoppers were collected from Zhejiang Province, China, and maintained under experimental conditions for more than 10 generations. The insects were reared on rice seedlings (Zhonghua 11 rice variety) at 25 ± 1°C under a 14:10 h (light: dark) photoperiod with a relative humidity of 60%–70%.

### Cell culture

Monkey (Vero) and human (HeLa and HEK293T) cell lines used for the MTT assay were cultured at 37°C with 20% (v/v) O_2_ and 5% (v/v) CO_2_. The cells were cultured in Dulbecco's modified Eagle medium (Gibco, 11965092) supplemented with 10% (v/v) fetal bovine serum (Gemini, 900‐108), 2 mM l‐glutamine (Gibco, 25030081), 50 µM 2‐mercaptoethanol (Thermo Fisher, 60‐24‐2), and 1% (v/v) penicillin−streptomycin (Gibco, 15140163). Sf9 cells that were transfected to express GFP‐tagged CDCs were cultured in incubators at 25°C with SIM SF Expression Medium (Sino Biological, MSF1). Leafhopper VCM cells were cultured in growth media [30].

### Genome assembly and quality assessment

PacBio long‐read and Illumina short‐read sequences were used for *de novo* assembly of the leafhopper genomes. For *Ne. cincticeps* and *R. dorsalis*, PacBio reads were assembled via the NextDenovo pipeline v2.3 [31], and the draft sequences were polished using NextPolish v1.2.4 [32] with the default parameters. To reduce assembly errors, PacBio reads were aligned to the assemblies using minimap2 v2.17 [33], and Polar Star (https://github.com/phasegenomics/polar_star) was applied to determine read depth. Then, outliers were found (100‐kb windows with a depth less than 0.75 times the average value or more than three times the average), and contigs were broken. For *Ne. nigropictus*, PacBio reads were first corrected with Canu v2.1 [34] and subsequently assembled using Wtdbg2 v2.5 [35], after which three rounds of polishing with Pilon v1.24 [36] were performed on the draft assembly via Illumina reads. Redundant contigs were removed using Purge Haplotigs v1.1.1 [37] based on the read coverage of the PacBio reads. The Hi‐C data were aligned to the draft assemblies with Juicer v1.6 [38], and the contigs were further anchored to superscaffolds using 3D‐DNA v180419 [39] based on the Hi‐C interaction results. Juicebox v1.11.8 (https://github.com/aidenlab/Juicebox) was used for visualizing the Hi‐C contact matrix and manually correcting misassembled chromosomes. To identify the sex chromosome, the Illumina reads were aligned to chromosome‐level assemblies using BWA‐MEM v0.7.17 [40], and the read coverage of 100‐kb windows was determined using SAMtools (v1.9) depth [41]. The completeness of the genome assemblies and annotations was evaluated against the Insecta_odb10 dataset, which included 1367 genes, using BUSCO v3.0.2 [42].

### Repeat and gene annotation

To identify transposable elements (TEs) in the leafhopper genomes, a species‐specific TE library was constructed by combining the *de novo* library predicted by RepeatModeler2 v2.0.1 [43] and LTR_retriever v2.8 [44] with the Repbase v19.06 [45], REXdb v3.0 [46], and Dfam v3.2 [47] databases. TE sequences were identified and classified via a homology search against the library using RepeatMasker v4.10 (http://www.repeatmasker.org). Tandem repeats were annotated using Tandem Repeats Finder v4.09 [48] and the MIcroSAtellite identification tool v2.1 [49].

Ab initio prediction, homology searches, and transcript‐based assembly were integrated for protein‐coding gene prediction. Augustus v2.4 [50] and SNAP v2006‐07‐28 [51] were used for ab initio gene prediction. The homologous sequences of the *Drosophila melanogaster* (BDGP6, GCA_000001215.4) [52], *Acyrthosiphon pisum* (GCA_000142985.2) [53], *Laodelphax striatella* (GCA_003335185.2) [54], *Nilaparvata lugens* (NilLug1.0, GCF_000757685.1) [55], and *Homalodisca vitripennis* (v0.5.3 (i5k), GCA_000696855.2) (10.15482/USDA. ADC/1410182) gene models were searched using GeMoMa v1.7 [56]. Transcriptome data were aligned to the reference genome using HISAT2 v2.0.4 [57], and genes were predicted using StringTie v1.2.3 [58], followed by GeneMarkS‐T v5.1 [59]. Trinity v2.11 [60] was used for the *de novo* assembly of transcripts, and genes were predicted by PASA v2.0.2 [61]. Final gene models were integrated using EVM v1.1.1 [62], and isoforms were updated by PASA. The functions and domains of protein‐coding genes were annotated based on sequence similarity. Gene Ontology (GO) terms and Kyoto Encyclopedia of Genes and Genomes pathways were annotated using eggNOG‐mapper v2 [63], and protein signatures were inferred by InterProScan v5.46 [64] against the InterPro database [65].

### Comparative genome analysis

Synteny among the three leafhopper genomes was detected and visualized by MCScan (Python version) [66]. To identify orthologous genes between leafhoppers and other insects, the orthogroups among *D. melanogaster*, *L. striatella*, *N. lugens*, *Oncopeltus fasciatus* (OGSv1.2 (I5K), GCA_000696205.2) [67], *H. vitripennis*, and the three leafhopper species were assigned using OrthoFinder v2.5.4 [68]. The species phylogeny was inferred from the CDSs of all single‐copy orthologs (907 kb in length). The maximum likelihood tree was reconstructed using IQ‐TREE v2.4 [69], with the best‐fitting nucleotide substitution model GTR + F + I + G4, and 100 bootstrap replicates were used. The species divergence times were estimated using MCMCTree [70] with the following parameters: burn‐in 5000000, sample 5000000, Sampfreq. 30, and correlation 1 (calibration points). Gene family and superfamily evolution was inferred using CAFÉ v4.2.1 [71] based on the time‐calibrated species tree. The gene numbers of each superfamily in the SUPERFAMILY database [72] were summarized according to the InterProScan v5.46 [64] results for all eight species.

### Evolution of key genes

Considering that the automatic annotation process may have led to errors, annotations of the focal gene families were manually checked according to the results of the homology search and transcriptome data alignments. For the homology method, the protein sequences of the *CDC* genes were aligned to the reference genome using GenBlastA v1.0.4 [73], and potential genes were predicted with GeneWise v2.4.1 [74]. To characterize the gene structure, SignalP v5.0 [75] and TMHMM v2.0 [76] were used to detect the signal peptide and transmembrane domain, respectively. Gene positions on the chromosomes were visualized using MG2C v2 [77].

To evaluate gene evolution rates, the CDSs of the homologs were aligned using PRANK v.170427 [78] with the translated alignment model, and the Ka/Ks ratios were calculated using KaKs Calculator v2.0 [79] with the LWL model. The branch‐site model in PAML v4.9 [70] was used to detect lineage‐specific positive selection signals, and the selected sites were inferred by Bayes empirical Bayes analysis.

Gene trees were constructed using RAxML v7.2.6 [80] with the amino acid substitution model PROTGAMMAJTT and 100 rapid bootstrap replicates based on the protein sequence alignments generated by MUSCLE v3.8.31 [81].

To determine phylogenetic relationships between the leafhopper *CDCs* and outgroup sequences, we used a hierarchical clustering method in accordance with previous studies, given that the dataset included prokaryote and eukaryote species and the sequences were highly divergent [21]. Specifically, protein sequences of the *CDC* family (Transporter Classification Database [TCDB] ID 1.C.12), the *MACPF* family (TCDB ID 1.C.39), and the *pleurotolysin B* family (TCDB ID 1.C.97.1) were downloaded from TCDB [82] (https://tcdb.org/). All available data for the *CDC* family and *pleurotolysin B* family were used, and only one sequence was selected to represent each cluster of the *MACPF* family. The sequences were pairwise aligned using BLASTP v2.2.26 [83], and for sequence pairs with an E‐value less than the cutoff (10), the distance between the two proteins was set to 100/bit. Otherwise, the distance was set to 10. The hclust function from the stats package in the program R was used for hierarchical cluster analysis based on the distance matrices, and the parameter “method” was set to average. The results were visualized with iTOL v6 [84].

To identify *CDC* homologs in other insect species, annotated protein datasets of insect genomes were downloaded from the NCBI RefSeq database (Table S3). Leafhopper CDC proteins were used as queries for BLASTP v2.2.26 [83] searches, and hits with an E‐value < 1e‐5 were considered potential *CDC* homologs. To trace the evolutionary history of *CDC* genes in Cicadellidae, all available Cicadellidae genome assemblies (as of August 2026) were downloaded from NCBI (Table S4). Due to the lack of genome annotation for some of these assemblies, we used BUSCO v3.0.2 [42] to predict the coding sequences of conserved insect genes (insecta_odb10 dataset) and used OrthoFinder [68] to obtain orthologous genes and construct the species tree. For the *CDC* gene family, as previously mentioned, GenBlastA v1.0.4 [73] and GeneWise v2.4.1 [74] were used for gene prediction.

RSCU was calculated using CodonW v1.4.4 [85] with default parameters and visualized using the pheatmap package.

### Protein structure prediction

The structure of the full‐length CDC protein was predicted using AlphaFold2 [23]. The prediction with the highest confidence was aligned to the crystal structure of perfringolysin O (PDB ID: 1PFO) [18] and visualized using PyMOL (The PyMOL Molecular Graphics System, Version 2.0, Schrödinger, LLC).

### Karyotyping assay

Testes were extracted from the abdomens of male adult leafhoppers, treated with hypotonic fluid (0.075 M KCl) for 15 min, and then fixed in Carnoy's fixative (ethanol: acetic acid = 3:1) for 2 h. Testicular follicles were separated on glass slides and stained in carbol fuchsin solution for 15 min. Then, the coverslips were pressed on. Images of cells in meiosis were captured under a microscope.

### DNA extraction and PCR

DNA was extracted using an Animal Genomic DNA Quick Extraction Kit for PCR Analysis (Beyotime, D0065S). *CDC* genes were amplified using 2× Es Taq MasterMix (Dye) (CWBIO, CW0690S). The primers used for PCR are listed in Table S5.

### RNA preparation and RT‐PCR and RT‐qPCR analyses

Total RNA was extracted using TRIzol Reagent (Invitrogen) and reverse‐transcribed with an M5 Super qPCR RT Kit containing gDNA remover (Mei5 Biotechnology, MF166). The cDNA obtained was used as the template for RT–PCR and qPCR. qPCR was performed using the CFX96 Real‐Time PCR Detection System (Bio‐Rad) with 2× Real‐time PCR Super Mix (SYBR Green, with anti‐Taq) (Mei5 Biotechnology, MF013). Relative transcript levels were calculated using the 2^−ΔΔC(t)^ method, with *Profilin* serving as the internal standard. The primers used for RT–qPCR are listed in Table S5.

### Gene cloning and plasmid construction

*RdCDC3* (*Id09G003230*), *RdCDC6* (*Id08G023250*), and *RdCDC9* (*Id09G000860*) sequences were used throughout this study. To clone the CDSs of *RdCDC3*, *6*, and *9*, total RNA was extracted from leafhoppers and used as a template for RT–PCR with the specific primers shown in Table S5. Full‐length *RdCDC3*, *6*, and *9* open reading frames (ORFs) were cloned and inserted into pFastHTB‐GFP for baculovirus expression in Sf9 cells. For yeast transformation, the ORFs of *RdCDC3*, *6*, and *9* were cloned and inserted into pGBK. For protein purification in *E. coli*, the ORFs of *RdCDC3*, *6*, and *9* were cloned and inserted into pGEX‐KG to express the GST‐tagged proteins RdCDC3, 6, and 9, respectively. All constructs used in this research were sequenced from both ends.

### Yeast activity assay

Yeast transformation and screening were performed according to the manufacturer's instructions (Clontech).

### Western blotting

Total proteins were extracted from different leafhopper tissues in SDS buffer [10% β‐mercaptoethanol, 100 mM Tris (pH 6.8), 20% glycerol, 4% SDS, and 0.2% bromophenol blue], separated via SDS‐PAGE, and transferred to nitrocellulose membranes. Protein accumulation was detected with the corresponding anti‐RdCDC3 (1:3000) polyclonal antibodies, and a goat anti‐rabbit IgG horseradish peroxidase conjugate (1:10,000) was used as a secondary antibody. Samples were subsequently incubated with Pierce ECL Plus chemiluminescent substrate before exposure.

### Baculovirus expression of RdCDCs in Sf9 cells

Recombinant baculovirus shuttle vectors expressing RdCDC3‐GFP, RdCDC6‐GFP, and RdCDC9‐GFP were constructed and transformed into *E. coli* DH10Bac cells. Recombinant bacmid DNA with the expected constructs was transfected into Sf9 cells using Cellfectin Reagent (Invitrogen) [86]. P1 recombinant baculovirus stock was collected after 5 days (>10^6^ pfu/mL). The virus was amplified by infecting Sf9 cells, after which the P2 recombinant baculovirus stock was collected (>10^7^ pfu/mL). Finally, sufficient virus‐infected Sf9 cells were collected at 2 dpi to detect the target proteins by western blotting or immunofluorescence.

### MST assay

To detect the ability of the *RdCDCs* to bind cholesterol, MST experiments were performed using a Monolith NT.115 (Blue/Red) instrument (NanoTemper Technologies GmbH) as previously described [87, 88]. Lysates of Sf9 cells expressing RdCDC3‐GFP, RdCDC6‐GFP, and RdCDC9‐GFP (above) were used as the sources of fluorescently labeled RdCDCs, whereas the concentration of cholesterol varied. After 15 min of incubation, the samples were loaded into MST standard glass capillaries. Measurements were performed at 22°C in MST buffer containing 10 mM Na‐phosphate buffer (pH 7.4), 1 mM MgCl_2_, 3 mM KCl, 150 mM NaCl, and 0.05% Tween‐20 to provide the optimal fluorescence intensity using a light‐emitting diode (LED) source with 40% LED power and 20% MST power on the MST machine. The Hill model was used to evaluate binding affinity, and each data point represents the mean ΔFnorm value from four independent experiments. Data were analyzed using NanoTemper analysis and MO.Affinity.

### Protein purification

RdCDCs were recombinantly expressed with an N‐terminal GST tag in the pGEX‐4T vector in *E. coli* strain BL21 (DE3) after 18 h at 16°C. Bacterial lysates were applied to GSTrap columns (GE Healthcare) equilibrated with PB buffer (200 mM NaCl, 20 mM Tris‐HCl, pH 7.5, and 10% [v/v] glycerol). The bound proteins were eluted using PB buffer containing 10 mM reduced glutathione (NEB).

### Cell viability assay (MTT assay)

Cells were counted and plated in 96‐well microplates overnight at a concentration of 1 × 10^4^/mL. Then, the cells were subjected to twofold serial dilutions of the RdCDCs in medium for 24 h at 37°C. Next, 100 µl of MTT diluted in serum‐free medium was added to each well, and the cells were incubated for 4 h at 37°C [20]. A total of 100 µL of solubilization solution (dimethyl sulfoxide) was added to each well, and the plate was incubated for an additional 10 min at room temperature, after which the absorbance of the generated formazan was measured at 493 nm using a microplate reader (BioTek). Cell viability curves were analyzed using Origin software (OriginPro 2020). The RdCDC concentration that resulted in 50% cell viability was defined as the IC_50_ value. Data are presented as the mean ± standard error of the mean (SEM) from three independent biological replicates.

### GUV preparation

To prepare GUVs containing different cholesterol concentrations, 0.05 mg of lipids were mixed at the desired ratios: 1,2‐dioleoyl‐sn‐glycero‐3‐phosphocholine (DOPC): 1,2‐dioleoyl‐sn‐glycero‐3‐phosphoethanolamine (DOPE) = 4:3, DOPC: DOPE: cholesterol = 4:3:1, or DOPC: DOPE: cholesterol = 4:3:2 for GUVs containing 0%, 10%, or 20% cholesterol, respectively. A total of 0.025% 18:1 Liss Rhod PE was added to fluorescently label the GUVs. GUVs were then prepared via the electroformation method using Vesicle Prep‐Pro (Nanion). Indium tin oxide (ITO)‐covered glass slides were gently cleaned with liquid soap, followed by three washes with distilled water and ethanol before use. A spacer (rubber ring) was attached to the ITO‐covered side of the slide to create a confined chamber. The lipid mixture was then deposited onto the confined glass surface and air‐dried to form a lipid film. The chamber was gently filled with 280 µL of 300 mM sorbitol, and electroformation was then carried out at 0.24 V and 9.9 Hz for 90 min at 37°C. The solution containing GUVs was collected and stored at 4°C before use.

### GUV perforation assay

The GUV perforation assay was performed using a Nikon A1 HD25 confocal microscope. Images were acquired using a 60× objective at 1024 × 1024 pixels. GUVs were diluted with 10 μg/mL FITC‐dextran (10 kDa). Three droplets of diluted GUVs were separately deposited on a glass‐bottom confocal chamber (Cellvis) and allowed to settle for 0.5–1 h before imaging. A three‐phase time‐lapse imaging protocol was used to capture the dynamics of perforation. In the first phase, three images of GUVs before the addition of protein were acquired at 30‐s intervals. The second phase was a 60‐s pause without acquisition, during which RdCDCs or PLO were added to the GUV droplets. Three different conditions could be performed in a single trial. The third phase was a 15‐min time‐lapse video monitoring the leakage or deformation of GUVs after protein addition.

Data processing was performed using NIS‐Elements software. First, alignment was performed using NIS‐Elements to correct XY motions. Then, regions of interest (ROIs) were defined in areas corresponding to the lumen of individual GUVs. Three reference ROIs were defined in GUV‐free spaces. Finally, the fluorescence intensity of FITC‐dextran over time was measured for all ROIs, and the results were normalized using the values of the reference ROIs. The final results were plotted using GraphPad Prism.

### Microinjection of dsRNA or RdCDC proteins into insects

Fragments were amplified by RT‐PCR, and the dsRNA specific to each leafhopper *CDC* and *GFP* was synthesized using a T7 RiboMAX Express RNAi Kit (Promega) following the manufacturer's instructions. Adult leafhoppers were injected with 1.5 µg/µL dsRNA via the thorax using a Nanoliter 2000 injector (World Precision Instruments). GST‐RdCDCs were purified using a previously described method. Adult *M. discolor* were injected with 1.5 µg of protein via the thorax using a Nanoliter 2000 injector (World Precision Instruments).

### Cell and egg immunofluorescence

Leafhopper eggs were carefully punctured near the egg cap, and Sf9 and VCM cells were seeded onto cell culture dishes and incubated with the GST‐RdCDC3, GST‐RdCDC6, GST‐RdCDC9, and GST proteins. Then, the cells were fixed with 4% paraformaldehyde (w/v) at room temperature for 20 min or 4°C overnight. The cells or eggs were washed three times with phosphate‐buffered saline (PBS), incubated for 10 min (30 min for eggs) after the addition of Triton X‐100 (final concentration, 0.5%), blocked with 5% bovine serum albumin diluted in PBS for 1 h, and incubated with primary antibodies against GST or RdCDC3 (1:100 dilution) for 1 h and fluorescently labeled secondary antibodies for 1 h. The slides were mounted using antifade mounting medium with 4',6‐diamidino‐2‐phenylindole (DAPI; Meilunbio, MA0222‐1). Fluorescence images were captured with an LSM 710 confocal microscope. The images were analyzed using ImageJ.

### Cryosection immunogold labeling

The eggs collected from the rice seedlings were fixed via high‐pressure freezing (HPF, Leica EM HPM100) with 5% sucrose as the cryoprotectant. Approximately 1–2 mL of acetone was added to an aluminum can, which was placed in liquid nitrogen until the acetone froze to form a solid. The frozen sample planchette was removed and placed on the aluminum with precooled tweezers. Freeze‐substitution was carried out as follows with 0.4% (w/v) uranyl acetate in acetone. First, the mixture was incubated at −85°C for 24 h, the temperature was slowly increased to −50°C over 5 h, and the mixture was rinsed in ethanol three times for an additional 2 h. Next, the temperature was slowly increased to −35°C and kept at this temperature for 48 h, after which the solution was changed to HM20 resin (Electron Microscopy Sciences, 14340) with gradually increasing concentrations (50%, 75% [v/v] in ethanol, 100%, 100%, 100%). Finally, the resin was polymerized at −35°C for 48 h. The sample was then cut into 80‐nm sections and placed on 100‐mesh nickel mesh. The sections were blocked with PBS containing 0.2% gelatin and 0.1% acetylated bovine serum albumin (BSA‐c) for 10 min. The primary antibody (diluted 100× in blocking solution) was added to each coverslip for 30 min of incubation at room temperature. Then, the samples were washed with PBS containing 0.1% BSA‐c for 2 min, and this process was repeated five times. Subsequently, sections were incubated with immunolabeled CMC‐Protein A‐gold (10 nm protein A‐gold; diluted 1:50) (Abcam) for 25 min and washed with PBS containing 0.1% BSA‐c 5 times for 2 min each. The sections were subsequently placed on copper wire mesh for observation using a JEOL JEM‐1400Flash (HC) transmission electron microscope (Japan).

### Feeding test with a natural enemy

To investigate the potential effects of leafhopper CDC proteins on the feeding of leafhopper predators, we conducted a feeding behavior experiment using *M. discolor* ladybugs, which were starved for 2 h before the experiments. A total of 2 mg of *E. kuehniella* eggs and *B. campestris* pollen were mixed with 10 µg of GST or purified GST‐RdCDC3/GST‐RdCDC9 proteins. The two kinds of food were positioned on each side of a Petri dish, which was 5.5 cm in diameter. A ladybug was placed into the Petri dish and allowed to move freely for 4 h. A light box was used for illumination, and a Sony FDR‐AX60 video recorder (1920 pixels × 1080 pixels @ 25 fps) was used to capture videos. The videos were downsampled to 1 fps and analyzed using OpenCV modules in Python to trace the position of the ladybug in each frame and count the duration of contact between the ladybug and the food as the feeding time.

### Quantitative metagenomic sequencing

Total DNA was extracted from the gut tissue of leafhoppers that had been injected with *dsGFP* or *dsRdCDC9* RNAs. Synthetic spike‐in sequences (Genesky Biotechnologies Inc.) with known and gradient copy numbers were added to the DNA, followed by library construction and Illumina sequencing, according to the manufacturer's instructions.

Raw reads were trimmed using Trimmomatic v0.39. Clean reads of each sample were first aligned to the spike‐in sequences using Bowtie2 v2.5.4 [89], and the standard curve was fitted as:

$$\log_{10}\;\left(\text{copies}\right)=a\times \log_{10}\;\left(\text{read counts}/\text{sequence length}\right)+b.$$

Clean data without spike‐in reads were assembled using MEGAHIT v1.2.9 [90] with the parameter: ‐‐k‐list 27, 47, 67, 87, 107, 127, 141. Contigs longer than 500 bp in all samples were combined, and redundant contigs were removed using MMseqs2 (release 17) [91] with an identity threshold of 95% and a coverage threshold of 90%. Prodigal v2.6.3 [92] was used for gene structure prediction with the parameter: ‐p meta, and redundant genes were removed as mentioned above. The taxonomy was annotated using CAT_pack software v6.0 [93] with the reference database of bacteria, archaea, fungi, and viruses. Genes associated with virulence factors were annotated by searching the VFDB [94] using DIAMOND v2.1 [95] (blastp ‐‐mid‐sensitive ‐‐id 80), and the top hit was adopted.

Clean reads were aligned to the nonredundant gene sets using the local mode of Bowtie2 v2.5.4 [89] (‐‐local ‐‐very‐sensitive‐local ‐‐score‐min G,20,6). Absolute abundances were calculated as:

absolute abundances (copies/ng)=10^(a×log10(read counts/sequence length)+b)/input DNA mass.

Downstream analyses were performed in R based on the abundance matrices of OTUs or gene categories. The α‐diversity index, Bray–Curtis distance, PERMANOVA test, and ANOSIM test were calculated using the vegan package. PCoA was performed using the “cmdscale” function. PLS‐DA was performed using the “plsda” function of the “mixOmics” package. Student's *t*‐test and LEfSe [96] were used to identify differentially abundant taxa. Differences in the absolute abundances of VFDB categories between groups were assessed using the Wilcoxon test.

### Statistical analysis

Data are presented as the mean ± SEM. The number of biological replicates is indicated in the legends. Differences in gene expression levels (Figure 4A), M. *discolor* body weight (Figure 4J), and leafhopper body weight (Figure 5B) between two independent groups were assessed using two‐tailed unpaired Student's *t* tests.

The feeding time of *M. discolor* in Figure 4H was analyzed using an LMM, with food type, position, and their interaction included as fixed effects and individual identity included as a random intercept to account for repeated measurements within individuals. The model was established as:

Feeding time ~food type×food position+(1|identity).

The statistical significance of the fixed effects was assessed using Type III *F* tests with Satterthwaite's method.

The percent weight gain of *M. discolor* in Figure 4I was analyzed using an LMM, with CDC protein injection condition, time after injection, and their interaction included as fixed effects and individual identity included as a random intercept to account for repeated measurements. The model was established as:

Percent weight gain ~condition×time+(1|identity).

The statistical significance of the fixed effects was assessed using Type III *F* tests with Satterthwaite's method.

Post hoc pairwise comparisons between injection conditions at each time point were performed based on estimated marginal means (EMMs), with *p* values adjusted for multiple comparisons using Tukey's method.

The body weight of *M. discolor* in Figure 4K was analyzed using an LMM, with egg condition, time, sex of *M. discolor*, and their interaction included as fixed effects and individual identity included as a random intercept to account for repeated measurements. The model was established as:

Body weight ~condition×time×sex+(1|identity).

The statistical significance of the fixed effects was assessed using Type III *F* tests with Satterthwaite's method.

Post hoc pairwise comparisons between egg conditions at each time point within each sex were performed based on EMMs, with *p* values adjusted for multiple comparisons using Tukey's method.

Analyses were performed in R using the “lme4,” “lmerTest,” and “emmeans” packages.

The survival rates of leafhoppers in Figure 5C were analyzed using a GLM with a quasibinomial error distribution and a logit link function to account for overdispersion. The numbers of surviving and dead individuals were included as the response, with the treatment group as the explanatory variable. Statistical significance was assessed using an *F* test.

Leafhopper offspring numbers in Figure 5D were analyzed using a GLM with a Poisson error distribution and a log link function, with treatment as the explanatory variable. The statistical significance of the treatment effect was assessed using a likelihood‐ratio *χ* ^2^ test.

## AUTHOR CONTRIBUTIONS

**Zhenjia Zhang**: Conceptualization; methodology; software; data curation; investigation; validation; formal analysis. **Yubo Zhang**: Conceptualization; software; data curation; formal analysis. **Kun Zhao**: Methodology; software; formal analysis. **Guangyuan Cheng**: Validation; formal analysis. **Zhihao Lu**: Validation; formal analysis. **Dongju Wang**: Validation. **Yiqun Liu**: Methodology. **Jianguo Wu**: Validation. **Liying Wang**: Investigation. **Lisheng Zhang**: Methodology. **Xinhai Ye**: Formal analysis. **Li Yu**: Formal analysis. **Wei Zhang**: Formal analysis; conceptualization; writing—original draft. **Yi Li**: Funding acquisition; writing—original draft; project administration; visualization; resources; supervision; writing—review and editing.

## CONFLICT OF INTEREST STATEMENT

The authors declare no conflicts of interest.

## ETHICS STATEMENT

No animals or humans were involved in this study.

## Supporting information


**Figure S1:** Chromosome‐level genome assembly and karyotyping analysis of *Ne. cincticeps*, *Ne. nigropictus*, and *R. dorsalis*.
**Figure S2:** Distributions and gene structures of *CDCs* in the three leafhopper species.
**Figure S3:** Gene structures of *CDCs* in *R. dorsalis*.
**Figure S4:** Protein similarity clusters of the *MACPF/CDC* superfamily and the selection signals of leafhopper *CDCs*.
**Figure S5:** Phylogeny of *CDC* genes in the family Cicadellidae.
**Figure S6:** Ka/Ks analysis of *CDC* genes in the family Cicadellidae.
**Figure S7:** Sequence alignments of motifs related to cholesterol recognition and binding and protein structure predictions.
**Figure S8:** Determination of HEK293T and insect Sf9 cell viability.
**Figure S9:** GUV assay of RdCDC3, 6, 9, and PLO.
**Figure S10:** *RdCDC* RNAi in leafhoppers.
**Figure S11:** The diversity and function of the leafhopper gut microbiome.

**Table S1:** Statistical comparison of draft genome assemblies among three leafhoppers.
**Table S2:** Statistical comparison of genome annotations among three leafhoppers.
**Table S3:** Number of *CDC* genes in insect genomes from the RefSeq database.
**Table S4:** Distribution of *CDC* genes within the family Cicadellidae.
**Table S5:** Primers used in this study.

**Video S1:** GUV perforation induced by different RdCDC proteins.

**Video S2:** GUV perforation at varying cholesterol contents.

**Video S3:** GUV perforation induced by RdCDC and bacterial CDC proteins.

## Data Availability

Raw PacBio sequencing, Illumina sequencing, and Hi‐C sequencing data were obtained from the National Center for Biotechnology Information (NCBI) under accession number PRJNA1008063 (https://www.ncbi.nlm.nih.gov/bioproject/PRJNA1008063). The raw gut microbiome sequencing data of *R. dorsalis* have been deposited in the NCBI under accession number PRJNA1529909 (https://www.ncbi.nlm.nih.gov/bioproject/PRJNA1529909). Scripts used for data analysis and visualization are available on GitHub (https://github.com/YuboZhangPKU/CDC_Evo). Supplementary materials (figures, tables, videos, graphical abstract, slides, Chinese translated version, and updated materials) may be found in the online DOI or iMeta Science http://www.imeta.science/.

## References

[1] Santos, M. Emília , Augustin Le Bouquin , Antonin J. J. Crumière , and Abderrahman Khila . 2017. “Taxon‐Restricted Genes at the Origin of a Novel Trait Allowing Access to a New Environment.” Science 358: 386–390. 10.1126/science.aan2748 29051384

[2] Panfilio, Kristen A. , and David R. Angelini . 2018. “By Land, Air, and Sea: Hemipteran Diversity through the Genomic Lens.” Current Opinion in Insect Science 25: 106–115. 10.1016/j.cois.2017.12.005 29602356

[3] Prud'homme, Benjamin , Caroline Minervino , Mélanie Hocine , Jessica D. Cande , Aïcha Aouane , Héloïse D. Dufour , Victoria A. Kassner , and Nicolas Gompel . 2011. “Body Plan Innovation in Treehoppers Through the Evolution of an Extra Wing‐Like Appendage.” Nature 473: 83–86. 10.1038/nature09977 21544145

[4] Erb, Matthias , and Philippe Reymond . 2019. “Molecular Interactions between Plants and Insect Herbivores.” Annual Review of Plant Biology 70: 527–557. 10.1146/annurev-arplant-050718-095910 30786233

[5] Dietrich, Christopher H. , Dmitry A. Dmitriev , Daniela M. Takiya , M. Jared Thomas , Michael D. Webb , James N. Zahniser , and Yalin Zhang . 2022. “Morphology‐Based Phylogenetic Analysis of Membracoidea (Hemiptera: Cicadomorpha) With Placement of Fossil Taxa and Description of a New Subfamily.” Insect Systematics and Diversity 6: 7. 10.1093/isd/ixac021

[6] Bai, Yuechen , Caiqiong Yang , Rayko Halitschke , Christian Paetz , Danny Kessler , Konrad Burkard , Emmanuel Gaquerel , Ian T. Baldwin , and Dapeng Li . 2022. “Natural History‐Guided Omics Reveals Plant Defensive Chemistry against Leafhopper Pests.” Science 375: eabm2948. 10.1126/science.abm2948 35113706

[7] Jia, Dongsheng , Qianzhuo Mao , Yong Chen , Yuyan Liu , Qian Chen , Wei Wu , Xiaofeng Zhang , et al. 2017. “Insect Symbiotic Bacteria Harbour Viral Pathogens for Transovarial Transmission.” Nature Microbiology 2: 17025. 10.1038/nmicrobiol.2017.25 28263320

[8] Huang, Yu , Jialin Yang , Xi Sun , Jiahao Li , Xiaoqiang Cao , Shengze Yao , Yanhong Han , et al. 2025. “Perception of Viral Infections and Initiation of Antiviral Defence in Rice.” Nature 641: 173–181. 10.1038/s41586-025-08706-8 PMC12043510 40074903

[9] Husnik, Filip , Naruo Nikoh , Ryuichi Koga , Laura Ross , Rebecca P. Duncan , Manabu Fujie , Makiko Tanaka , et al. 2013. “Horizontal Gene Transfer From Diverse Bacteria to an Insect Genome Enables a Tripartite Nested Mealybug Symbiosis.” Cell 153: 1567–1578. 10.1016/j.cell.2013.05.040 23791183

[10] Nouri, Shahideh , Emilyn E. Matsumura , Yen‐Wen Kuo , and Bryce W. Falk . 2018. “Insect‐Specific Viruses: From Discovery to Potential Translational Applications.” Current Opinion in Virology 33: 33–41. 10.1016/j.coviro.2018.07.006 30048906

[11] Li, Zheng , Yiyuan Li , Allen Z. Xue , Vy Dang , V. Renee Holmes , J. Spencer Johnston , Jeffrey E. Barrick , and Nancy A. Moran . 2022. “The Genomic Basis of Evolutionary Novelties in a Leafhopper.” Molecular Biology and Evolution 39: msac184. 10.1093/molbev/msac184 PMC9450646 36026509

[12] Gasmi, Laila , Edyta Sieminska , Shohei Okuno , Rie Ohta , Cathy Coutu , Mohammad Vatanparast , Stephanie Harris , et al. 2021. “Horizontally Transmitted Parasitoid Killing Factor Shapes Insect Defense to Parasitoids.” Science 373: 535–541. 10.1126/science.abb6396 34326235

[13] Guo, Zhongxin , Yang Li , and Shou‐Wei Ding . 2019. “Small RNA‐Based Antimicrobial Immunity.” Nature Reviews Immunology 19: 31–44. 10.1038/s41577-018-0071-x 30301972

[14] Kovacs, Jennifer L. , Candice Wolf , Dené Voisin , and Seth Wolf . 2017. “Evidence of Indirect Symbiont Conferred Protection Against the Predatory Lady Beetle *Harmonia axyridis* in the Pea Aphid.” BMC Ecology 17: 26. 10.1186/s12898-017-0136-x PMC5504669 28693550

[15] Perreau, Julie , and Nancy A. Moran . 2022. “Genetic Innovations in Animal‐Microbe Symbioses.” Nature Reviews Genetics 23: 23–39. 10.1038/s41576-021-00395-z PMC8832400 34389828

[16] Billington, S . 2000. “Thiol‐Activated Cytolysins: Structure, Function and Role in Pathogenesis.” FEMS Microbiology Letters 182: 197–205. 10.1016/S0378-1097(99)00536-4 10620666

[17] Johnstone, Bronte A. , Riya Joseph , Michelle P. Christie , Craig J. Morton , Conall McGuiness , James C. Walsh , Till Böcking , Rodney K. Tweten , and Michael W. Parker . 2022. “Cholesterol‐Dependent Cytolysins: The Outstanding Questions.” IUBMB Life 74: 1169–1179. 10.1002/iub.2661 PMC9712165 35836358

[18] Rossjohn, Jamie , Susanne C. Feil , William J. McKinstry , Rodney K. Tweten , and Michael W. Parker . 1997. “Structure of a Cholesterol‐Binding, Thiol‐Activated Cytolysin and a Model of Its Membrane Form.” Cell 89: 685–692. 10.1016/S0092-8674(00)80251-2 9182756

[19] Shatursky, Oleg , Alejandro P. Heuck , Laura A. Shepard , Jamie Rossjohn , Michael W. Parker , Arthur E. Johnson , and Rodney K. Tweten . 1999. “The Mechanism of Membrane Insertion for a Cholesterol‐Dependent Cytolysin.” Cell 99: 293–299. 10.1016/S0092-8674(00)81660-8 10555145

[20] Xiong, Xiaozhe , Songhai Tian , Pan Yang , Francois Lebreton , Huan Bao , Kuanwei Sheng , Linxiang Yin , et al. 2022. “Emerging *Enterococcus* Pore‐Forming Toxins With MHC/HLA‐I as Receptors.” Cell 185: 1157–1171.e22. 10.1016/j.cell.2022.02.002 PMC8978092 35259335

[21] Moreno‐Hagelsieb, Gabriel , Bennett Vitug , Arturo Medrano‐Soto , and Milton H. Saier Jr. 2017. “The Membrane Attack Complex/Perforin Superfamily.” Microbial Physiology 27: 252–267. 10.1159/000481286 PMC5796659 29145176

[22] Reboul, Cyril F. , James C. Whisstock , and Michelle A. Dunstone . 2016. “Giant MACPF/CDC Pore Forming Toxins: A Class of Their Own.” Biochimica et Biophysica Acta (BBA) ‐ Biomembranes 1858: 475–486. 10.1016/j.bbamem.2015.11.017 26607011

[23] Jumper, John , Richard Evans , Alexander Pritzel , Tim Green , Michael Figurnov , Olaf Ronneberger , Kathryn Tunyasuvunakool , et al. 2021. “Highly Accurate Protein Structure Prediction With AlphaFold.” Nature 596: 583–589. 10.1038/s41586-021-03819-2 PMC8371605 34265844

[24] Grose, Carissa , Zoe Putman , and Dominic Esposito . 2021. “A Review of Alternative Promoters for Optimal Recombinant Protein Expression in Baculovirus‐Infected Insect Cells.” Protein Expression and Purification 186: 105924. 10.1016/j.pep.2021.105924 PMC8266756 34087362

[25] Shanker, C. , M. Mohan , M. Sampathkumar , Ch Lydia , and G. Katti . 2013. “Functional Significance of *Micraspis discolor* (F.) (Coccinellidae: Coleoptera) in Rice Ecosystem.” Journal of Applied Entomology 137: 601–609. 10.1111/jen.12035

[26] Zahniser, James N. , and Christopher H. Dietrich . 2010. “Phylogeny of the Leafhopper Subfamily Deltocephalinae (Hemiptera: Cicadellidae) Based on Molecular and Morphological Data With a Revised Family‐Group Classification.” Systematic Entomology 35: 489–511. 10.1111/j.1365-3113.2010.00522.x

[27] Li, Yang , Zhiguo Liu , Chao Liu , Zheyi Shi , Lan Pang , Chuzhen Chen , Yun Chen , et al. 2022. “HGT Is Widespread in Insects and Contributes to Male Courtship in Lepidopterans.” Cell 185: 2975–2987.e10. 10.1016/j.cell.2022.06.014 PMC9357157 35853453

[28] Steenwyk, Jacob L. , Yuanning Li , Xiaofan Zhou , Xing‐Xing Shen , and Antonis Rokas . 2023. “Incongruence in the Phylogenomics Era.” Nature Reviews Genetics 24: 834–850. 10.1038/s41576-023-00620-x PMC11499941 37369847

[29] Liu, Chao , Yang Li , Yun Chen , Xue‐xin Chen , Jianhua Huang , Antonis Rokas , and Xing‐Xing Shen . 2023. “How Has Horizontal Gene Transfer Shaped the Evolution of Insect Genomes?” Environmental Microbiology 25: 642–645. 10.1111/1462-2920.16311 PMC10153070 36511824

[30] Kimura, Ikuo . 1984. “Establishment of New Cell Lines From Leafhopper Vector and Inoculation of Its Cell Monolayers With Rice Dwarf Virus.” Proceedings of the Japan Academy, Series B 60: 198–201. 10.2183/pjab.60.198

[31] Hu, Jiang , Zhuo Wang , Zongyi Sun , Benxia Hu , Adeola Oluwakemi Ayoola , Fan Liang , Jingjing Li , et al. 2024. “NextDenovo: An Efficient Error Correction and Accurate Assembly Tool for Noisy Long Reads.” Genome Biology 25: 107. 10.1186/s13059-024-03252-4 PMC11046930 38671502

[32] Hu, Jiang , Junpeng Fan , Zongyi Sun , and Shanlin Liu . 2020. “NextPolish: A Fast and Efficient Genome Polishing Tool for Long‐Read Assembly.” Bioinformatics 36: 2253–2255. 10.1093/bioinformatics/btz891 31778144

[33] Li, Heng . 2018. “Minimap2: Pairwise Alignment for Nucleotide Sequences.” Bioinformatics 34: 3094–3100. 10.1093/bioinformatics/bty191 PMC6137996 29750242

[34] Koren, Sergey , Brian P. Walenz , Konstantin Berlin , Jason R. Miller , Nicholas H. Bergman , and Adam M. Phillippy . 2017. “Canu: Scalable and Accurate Long‐Read Assembly Via Adaptive K‐Mer Weighting and Repeat Separation.” Genome Research 27: 722–736. 10.1101/gr.215087.116 PMC5411767 28298431

[35] Ruan, Jue , and Heng Li . 2020. “Fast and Accurate Long‐Read Assembly With wtdbg2.” Nature Methods 17: 155–158. 10.1038/s41592-019-0669-3 PMC7004874 31819265

[36] Walker, Bruce J. , Thomas Abeel , Terrance Shea , Margaret Priest , Amr Abouelliel , Sharadha Sakthikumar , Christina A. Cuomo , et al. 2014. “Pilon: An Integrated Tool for Comprehensive Microbial Variant Detection and Genome Assembly Improvement.” PLOS ONE 9: e112963. 10.1371/journal.pone.0112963 PMC4237348 25409509

[37] Roach, Michael J. , Simon A. Schmidt , and Anthony R. Borneman . 2018. “Purge Haplotigs: Allelic Contig Reassignment for Third‐Gen Diploid Genome Assemblies.” BMC Bioinformatics 19: 460. 10.1186/s12859-018-2485-7 PMC6267036 30497373

[38] Durand, Neva C. , Muhammad S. Shamim , Ido Machol , Suhas S. P. Rao , Miriam H. Huntley , Eric S. Lander , and Erez Lieberman Aiden . 2016. “Juicer Provides a One‐Click System for Analyzing Loop‐Resolution Hi‐C Experiments.” Cell Systems 3: 95–98. 10.1016/j.cels.2016.07.002 PMC5846465 27467249

[39] Dudchenko, Olga , Sanjit S. Batra , Arina D. Omer , Sarah K. Nyquist , Marie Hoeger , Neva C. Durand , Muhammad S. Shamim , et al. 2017. “De Novo Assembly of the *Aedes aegypti* Genome Using Hi‐C Yields Chromosome‐Length Scaffolds.” Science 356: 92–95. 10.1126/science.aal3327 PMC5635820 28336562

[40] Li, Heng , and Richard Durbin . 2009. “Fast and Accurate Short Read Alignment With Burrows‐Wheeler Transform.” Bioinformatics 25: 1754–1760. 10.1093/bioinformatics/btp324 PMC2705234 19451168

[41] Li, Heng , Bob Handsaker , Alec Wysoker , Tim Fennell , Jue Ruan , Nils Homer , Gabor Marth , Goncalo Abecasis , and Richard Durbin , 1000 Genome Project Data Processing Subgroup . 2009. “The Sequence Alignment/Map Format and SAMtools.” Bioinformatics 25: 2078–2079. 10.1093/bioinformatics/btp352 PMC2723002 19505943

[42] Waterhouse, Robert M. , Mathieu Seppey , Felipe A. Simão , Mosè Manni , Panagiotis Ioannidis , Guennadi Klioutchnikov , Evgenia V. Kriventseva , and Evgeny M. Zdobnov . 2018. “BUSCO Applications From Quality Assessments to Gene Prediction and Phylogenomics.” Molecular Biology and Evolution 35: 543–548. 10.1093/molbev/msx319 PMC5850278 29220515

[43] Flynn, Jullien M. , Robert Hubley , Clément Goubert , Jeb Rosen , Andrew G. Clark , Cédric Feschotte , and Arian F. Smit . 2020. “RepeatModeler2 for Automated Genomic Discovery of Transposable Element Families.” Proceedings of the National Academy of Sciences 117: 9451–9457. 10.1073/pnas.1921046117 PMC7196820 32300014

[44] Ou, Shujun , and Ning Jiang . 2018. “LTR_retriever: A Highly Accurate and Sensitive Program for Identification of Long Terminal Repeat Retrotransposons.” Plant Physiology 176: 1410–1422. 10.1104/pp.17.01310 PMC5813529 29233850

[45] Bao, Weidong , Kenji K. Kojima , and Oleksiy Kohany . 2015. “Repbase Update, a Database of Repetitive Elements in Eukaryotic Genomes.” Mobile DNA 6: 11. 10.1186/s13100-015-0041-9 PMC4455052 26045719

[46] Neumann, Pavel , Petr Novák , Nina Hoštáková , and Jiří Macas . 2019. “Systematic Survey of Plant LTR‐Retrotransposons Elucidates Phylogenetic Relationships of Their Polyprotein Domains and Provides a Reference for Element Classification.” Mobile DNA 10(1): 1. 10.1186/s13100-018-0144-1 PMC6317226 30622655

[47] Storer, Jessica , Robert Hubley , Jeb Rosen , Travis J. Wheeler , and Arian F. Smit . 2021. “The Dfam Community Resource of Transposable Element Families, Sequence Models, and Genome Annotations.” Mobile DNA 12: 2. 10.1186/s13100-020-00230-y PMC7805219 33436076

[48] Benson, G . 1999. “Tandem Repeats Finder: A Program to Analyze DNA Sequences.” Nucleic Acids Research 27: 573–580. 10.1093/nar/27.2.573 PMC148217 9862982

[49] Beier, Sebastian , Thomas Thiel , Thomas Münch , Uwe Scholz , and Martin Mascher . 2017. “MISA‐web: A Web Server for Microsatellite Prediction.” Bioinformatics 33: 2583–2585. 10.1093/bioinformatics/btx198 PMC5870701 28398459

[50] Stanke, Mario , Mark Diekhans , Robert Baertsch , and David Haussler . 2008. “Using Native and Syntenically Mapped cDNA Alignments to Improve De Novo Gene Finding.” Bioinformatics 24: 637–644. 10.1093/bioinformatics/btn013 18218656

[51] Korf, Ian . 2004. “Gene Finding in Novel Genomes.” BMC Bioinformatics 5: 59. 10.1186/1471-2105-5-59 PMC421630 15144565

[52] Hoskins, Roger A. , Joseph W. Carlson , Kenneth H. Wan , Soo Park , Ivonne Mendez , Samuel E. Galle , Benjamin W. Booth , et al. 2015. “The Release 6 Reference Sequence of the *Drosophila melanogaster* Genome.” Genome Research 25: 445–458. 10.1101/gr.185579.114 PMC4352887 25589440

[53] The International Aphid Genomics Consortium . 2010. “Genome Sequence of the Pea Aphid *Acyrthosiphon pisum* .” PLOS Biology 8: e1000313. 10.1371/journal.pbio.1000313 PMC2826372 20186266

[54] Zhu, Junjie , Feng Jiang , Xianhui Wang , Pengcheng Yang , Yanyuan Bao , Wan Zhao , Wei Wang , et al. 2017. “Genome Sequence of the Small Brown Planthopper, *Laodelphax striatellus* .” GigaScience 6: gix109. 10.1093/gigascience/gix109 PMC5740986 29136191

[55] Xue, Jian , Xin Zhou , Chuan‐Xi Zhang , Li‐Li Yu , Hai‐Wei Fan , Zhuo Wang , Hai‐Jun Xu , et al. 2014. “Genomes of the Rice Pest Brown Planthopper and Its Endosymbionts Reveal Complex Complementary Contributions for Host Adaptation.” Genome Biology 15: 521. 10.1186/s13059-014-0521-0 PMC4269174 25609551

[56] Keilwagen, Jens , Michael Wenk , Jessica L. Erickson , Martin H. Schattat , Jan Grau , and Frank Hartung . 2016. “Using Intron Position Conservation for Homology‐Based Gene Prediction.” Nucleic Acids Research 44: e89. 10.1093/nar/gkw092 PMC4872089 26893356

[57] Kim, Daehwan , Joseph M. Paggi , Chanhee Park , Christopher Bennett , and Steven L. Salzberg . 2019. “Graph‐Based Genome Alignment and Genotyping With HISAT2 and HISAT‐genotype.” Nature Biotechnology 37: 907–915. 10.1038/s41587-019-0201-4 PMC7605509 31375807

[58] Kovaka, Sam , Aleksey V. Zimin , Geo M. Pertea , Roham Razaghi , Steven L. Salzberg , and Mihaela Pertea . 2019. “Transcriptome Assembly from Long‐Read RNA‐Seq Alignments With StringTie2.” Genome Biology 20: 278. 10.1186/s13059-019-1910-1 PMC6912988 31842956

[59] Besemer, J . 2001. “GeneMarkS: A Self‐Training Method for Prediction of Gene Starts in Microbial Genomes. Implications for Finding Sequence Motifs in Regulatory Regions.” Nucleic Acids Research 29: 2607–2618. 10.1093/nar/29.12.2607 PMC55746 11410670

[60] Grabherr, Manfred G. , Brian J. Haas , Moran Yassour , Joshua Z. Levin , Dawn A. Thompson , Ido Amit , Xian Adiconis , et al. 2011. “Full‐Length Transcriptome Assembly From RNA‐Seq Data Without a Reference Genome.” Nature Biotechnology 29: 644–652. 10.1038/nbt.1883 PMC3571712 21572440

[61] Haas, B. J . 2003. “Improving the *Arabidopsis* Genome Annotation Using Maximal Transcript Alignment Assemblies.” Nucleic Acids Research 31: 5654–5666. 10.1093/nar/gkg770 PMC206470 14500829

[62] Haas, Brian J. , Steven L. Salzberg , Wei Zhu , Mihaela Pertea , Jonathan E. Allen , Joshua Orvis , Owen White , C. Robin Buell , and Jennifer R. Wortman . 2008. “Automated Eukaryotic Gene Structure Annotation Using EVidenceModeler and the Program to Assemble Spliced Alignments.” Genome Biology 9: R7. 10.1186/gb-2008-9-1-r7 PMC2395244 18190707

[63] Huerta‐Cepas, Jaime , Damian Szklarczyk , Davide Heller , Ana Hernández‐Plaza , Sofia K. Forslund , Helen Cook , Daniel R. Mende , et al. 2019. “eggNOG 5.0: A Hierarchical, Functionally and Phylogenetically Annotated Orthology Resource Based on 5090 Organisms and 2502 Viruses.” Nucleic Acids Research 47: D309–D314. 10.1093/nar/gky1085 PMC6324079 30418610

[64] Jones, Philip , David Binns , Hsin‐Yu Chang , Matthew Fraser , Weizhong Li , Craig McAnulla , Hamish McWilliam , et al. 2014. “InterProScan 5: Genome‐Scale Protein Function Classification.” Bioinformatics 30: 1236–1240. 10.1093/bioinformatics/btu031 PMC3998142 24451626

[65] Blum, Matthias , Hsin‐Yu Chang , Sara Chuguransky , Tiago Grego , Swaathi Kandasaamy , Alex Mitchell , Gift Nuka , et al. 2021. “The InterPro Protein Families and Domains Database: 20 Years on.” Nucleic Acids Research 49: D344–D354. 10.1093/nar/gkaa977 PMC7778928 33156333

[66] Tang, Haibao , John E. Bowers , Xiyin Wang , Ray Ming , Maqsudul Alam , and Andrew H. Paterson . 2008. “Synteny and Collinearity in Plant Genomes.” Science 320: 486–488. 10.1126/science.1153917 18436778

[67] Panfilio, Kristen A. , Iris M. Vargas Jentzsch , Joshua B. Benoit , Deniz Erezyilmaz , Yuichiro Suzuki , Stefano Colella , Hugh M. Robertson , et al. 2019. “Molecular Evolutionary Trends and Feeding Ecology Diversification in the Hemiptera, Anchored By the Milkweed Bug Genome.” Genome Biology 20: 64. 10.1186/s13059-019-1660-0 PMC6444547 30935422

[68] Emms, David M. , and Steven Kelly . 2019. “OrthoFinder: Phylogenetic Orthology Inference for Comparative Genomics.” Genome Biology 20: 238. 10.1186/s13059-019-1832-y PMC6857279 31727128

[69] Nguyen, Lam‐Tung , Heiko A. Schmidt , Arndt von Haeseler , and Bui Quang Minh . 2015. “IQ‐TREE: A Fast and Effective Stochastic Algorithm for Estimating Maximum‐Likelihood Phylogenies.” Molecular Biology and Evolution 32: 268–274. 10.1093/molbev/msu300 PMC4271533 25371430

[70] Yang, Z . 2007. “PAML 4: Phylogenetic Analysis by Maximum Likelihood.” Molecular Biology and Evolution 24: 1586–1591. 10.1093/molbev/msm088 17483113

[71] De Bie, Tijl , Nello Cristianini , Jeffery P. Demuth , and Matthew W. Hahn . 2006. “CAFE: A Computational Tool for the Study of Gene Family Evolution.” Bioinformatics 22: 1269–1271. 10.1093/bioinformatics/btl097 16543274

[72] Gough, Julian , Kevin Karplus , Richard Hughey , and Cyrus Chothia . 2001. “Assignment of Homology to Genome Sequences Using a Library of Hidden Markov Models That Represent All Proteins of Known Structure.” Journal of Molecular Biology 313: 903–919. 10.1006/jmbi.2001.5080 11697912

[73] She, Rong , Jeffrey S.‐C. Chu , Ke Wang , Jian Pei , and Nansheng Chen . 2009. “genBlastA: Enabling BLAST to Identify Homologous Gene Sequences.” Genome Research 19: 143–149. 10.1101/gr.082081.108 PMC2612959 18838612

[74] Birney, Ewan , Michele Clamp , and Richard Durbin . 2004. “GeneWise and Genomewise.” Genome Research 14: 988–995. 10.1101/gr.1865504 PMC479130 15123596

[75] Almagro Armenteros, José Juan , Konstantinos D. Tsirigos , Casper Kaae Sønderby , Thomas Nordahl Petersen , Ole Winther , Søren Brunak , Gunnar von Heijne , and Henrik Nielsen . 2019. “SignalP 5.0 Improves Signal Peptide Predictions Using Deep Neural Networks.” Nature Biotechnology 37: 420–423. 10.1038/s41587-019-0036-z 30778233

[76] Krogh, Anders , Björn Larsson , Gunnar von Heijne , and Erik L. L. Sonnhammer . 2001. “Predicting Transmembrane Protein Topology With a Hidden Markov Model: Application to Complete Genomes.” Journal of Molecular Biology 305: 567–580. 10.1006/jmbi.2000.4315 11152613

[77] Chao, Jiangtao , Zhiyuan Li , Yuhe Sun , Oluwaseun Olayemi Aluko , Xinru Wu , Qian Wang , and Guanshan Liu . 2021. “MG2C: A User‐Friendly Online Tool for Drawing Genetic Maps.” Molecular Horticulture 1: 16. 10.1186/s43897-021-00020-x PMC10514940 37789491

[78] Löytynoja, Ari . 2014. “Phylogeny‐Aware Alignment With PRANK.” In Multiple Sequence Alignment Methods, edited by David J. Russell , 155–170. Methods in Molecular Biology 1079 Totowa, NJ: Humana Press. 10.1007/978-1-62703-646-7_10 24170401

[79] Wang, Dapeng , Yubin Zhang , Zhang Zhang , Jiang Zhu , and Jun Yu . 2010. “KaKs_Calculator 2.0: A Toolkit Incorporating Gamma‐Series Methods and Sliding Window Strategies.” Genomics, Proteomics & Bioinformatics 8: 77–80. 10.1016/S1672-0229(10)60008-3 PMC5054116 20451164

[80] Stamatakis, Alexandros . 2014. “RAxML Version 8: A Tool for Phylogenetic Analysis and Post‐Analysis of Large Phylogenies.” Bioinformatics 30: 1312–1313. 10.1093/bioinformatics/btu033 PMC3998144 24451623

[81] Edgar, R. C . 2004. “MUSCLE: Multiple Sequence Alignment With High Accuracy and High Throughput.” Nucleic Acids Research 32: 1792–1797. 10.1093/nar/gkh340 PMC390337 15034147

[82] Saier, Milton H. , Vamsee S. Reddy , Gabriel Moreno‐Hagelsieb , Kevin J. Hendargo , Yichi Zhang , Vasu Iddamsetty , Katie Jing Kay Lam , et al. 2021. “The Transporter Classification Database (TCDB): 2021 Update.” Nucleic Acids Research 49: D461–D467. 10.1093/nar/gkaa1004 PMC7778945 33170213

[83] Altschul, Stephen F. , Warren Gish , Webb Miller , Eugene W. Myers , and David J. Lipman . 1990. “Basic Local Alignment Search Tool.” Journal of Molecular Biology 215: 403–410. 10.1016/S0022-2836(05)80360-2 2231712

[84] Letunic, Ivica , and Peer Bork . 2021. “Interactive Tree of Life (iTOL) v5: an Online Tool for Phylogenetic Tree Display and Annotation.” Nucleic Acids Research 49: W293–W296. 10.1093/nar/gkab301 PMC8265157 33885785

[85] Peden, John F . 1999. Analysis of Codon Usage. PhD diss. University of Nottingham. https://codonw.sourceforge.net/JohnPedenThesisPressOpt_water.pdf

[86] Zhou, Feng , Yingying Pu , Taiyun Wei , Huijun Liu , Wulan Deng , Chunhong Wei , Biao Ding , Toshihiro Omura , Yi Li . 2007. “The P2 Capsid Protein of the Nonenveloped Rice Dwarf Phytoreovirus Induces Membrane Fusion in Insect Host Cells.” Proceedings of the National Academy of Sciences of the United States of America 104: 19547–19552. 10.1073/pnas.0708946104 PMC2148326 18042708

[87] Casarotto, Plinio C. , Mykhailo Girych , Senem M. Fred , Vera Kovaleva , Rafael Moliner , Giray Enkavi , Caroline Biojone , et al. 2021. “Antidepressant Drugs Act By Directly Binding to TRKB Neurotrophin Receptors.” Cell 184: 1299–1313.e19. 10.1016/j.cell.2021.01.034 PMC7938888 33606976

[88] Yang, Zhirui , Yu Huang , Jialin Yang , Shengze Yao , Kun Zhao , Donghui Wang , Qingqing Qin , et al. 2020. “Jasmonate Signaling Enhances RNA Silencing and Antiviral Defense in Rice.” Cell Host & Microbe 28: 89–103.e8. 10.1016/j.chom.2020.05.001 32504578

[89] Langmead, Ben , and Steven L. Salzberg . 2012. “Fast Gapped‐Read Alignment With Bowtie 2.” Nature Methods 9: 357–359. 10.1038/nmeth.1923 PMC3322381 22388286

[90] Li, Dinghua , Chi‐Man Liu , Ruibang Luo , Kunihiko Sadakane , and Tak‐Wah Lam . 2015. “MEGAHIT: An Ultra‐Fast Single‐Node Solution for Large and Complex Metagenomics Assembly via Succinct de Bruijn Graph.” Bioinformatics 31: 1674–1676. 10.1093/bioinformatics/btv033 25609793

[91] Steinegger, Martin , and Johannes Söding . 2017. “MMseqs. 2 Enables Sensitive Protein Sequence Searching for the Analysis of Massive Data Sets.” Nature Biotechnology 35: 1026–1028. 10.1038/nbt.3988 29035372

[92] Hyatt, Doug , Gwo‐Liang Chen , Philip F. LoCascio , Miriam L. Land , Frank W. Larimer , and Loren J. Hauser . 2010. “Prodigal: Prokaryotic Gene Recognition and Translation Initiation Site Identification.” BMC Bioinformatics 11: 119. 10.1186/1471-2105-11-119 PMC2848648 20211023

[93] von Meijenfeldt, F. A. Bastiaan , Ksenia Arkhipova , Diego D. Cambuy , Felipe H. Coutinho , Bas E. Dutilh . 2019. “Robust Taxonomic Classification of Uncharted Microbial Sequences and Bins With CAT and BAT.” Genome Biology 20: 217. 10.1186/s13059-019-1817-x PMC6805573 31640809

[94] Zhou, Siyu , Bo Liu , Dandan Zheng , Lihong Chen , and Jian Yang . 2025. “VFDB 2025: An Integrated Resource for Exploring Anti‐Virulence Compounds.” Nucleic Acids Research 53: D871–D877. 10.1093/nar/gkae968 PMC11701737 39470738

[95] Buchfink, Benjamin , Klaus Reuter , and Hajk‐Georg Drost . 2021. “Sensitive Protein Alignments at Tree‐of‐Life Scale Using DIAMOND.” Nature Methods 18: 366–368. 10.1038/s41592-021-01101-x PMC8026399 33828273

[96] Segata, Nicola , Jacques Izard , Levi Waldron , Dirk Gevers , Larisa Miropolsky , Wendy S. Garrett , and Curtis Huttenhower . 2011. “Metagenomic Biomarker Discovery and Explanation.” Genome Biology 12: R60. 10.1186/gb-2011-12-6-r60 PMC3218848 21702898

