# Supplementary material for: Domesticated bacterial origin genes regulate homeostasis of the insect gut microbiome and empower adaptation
Source: Imeta. 2026 Oct 15;5(5):e70179. doi: 10.1002/imt2.70179 (PMC13670155; doi:10.1002/imt2.70179)
Supplement: Supplementary file 1 [file IMT2-5-e70179-s005.docx]

**Supporting Information to**

**Domesticated bacterial origin genes regulate homeostasis of the insect gut microbiome and empower adaptation**

**Running title:** Domestication of horizontally transferred genes promotes insect adaptation

Zhenjia Zhang^1,2,3,#^, Yubo Zhang^2,#^, Kun Zhao^2,#^, Guangyuan Cheng^1^, Zhihao Lu^2^, Dongju Wang^4^, Yiqun Liu^5^, Jianguo Wu^1^, Liying Wang^2^, Lisheng Zhang^6^, Xinhai Ye^7^, Li Yu^4^, Wei Zhang^2,8,9,10,11,12,*^, Yi Li^1,12,*^

^1^State Key Laboratory of Agricultural and Forestry Biosecurity, Vector-borne Virus Research Center, Fujian Agriculture and Forestry University, Fuzhou 350002, China.

^2^Laboratory of Gene Function and Modulation Research, School of Life Sciences, Peking University, Beijing 100871, China.

^3^College of Grassland Science and Technology, China Agricultural University, Beijing 100193, China.

^4^The State Key Laboratory of Membrane Biology, Tsinghua University-Peking University Joint Centre for Life Sciences, Beijing Frontier Research Center for Biological Structure, School of Life Sciences, Tsinghua University, Beijing 100084, China.

^5^National Center for Protein Science and Core Facilities at School of Life Sciences, Peking University, Beijing 100871, China.

^6^Key Laboratory of Natural Enemy Insects of Ministry of Agriculture and Rural Affairs, State Key Laboratory for Biology of Plant Diseases and Insect Pests, Institute of Plant Protection, Chinese Academy of Agricultural Sciences, Beijing 100193, China.

^7^College of Advanced Agriculture Sciences, Zhejiang A&F University, Hangzhou 311300, China

^8^Peking-Tsinghua Center for Life Sciences, Academy for Advanced Interdisciplinary Studies, Peking University, Beijing 100871, China.

^9^Institute of Ecology, Peking University, Beijing 100871, China.

^10^Peking University Chengdu Academy for Advanced Interdisciplinary Biotechnologies, Chengdu 610213, China.

^11^Medog Biodiversity Observation and Research Station of Xizang Autonomous Region, Nyingchi 860711, China.

^12^Lead contact

^#^These authors contributed equally to this work: Zhenjia Zhang, Yubo Zhang, Kun Zhao

*Correspondence: [liyi@pku.edu.cn](mailto:liyi@pku.edu.cn) (Yi Li), [weizhangvv@pku.edu.cn](mailto:weizhangvv@pku.edu.cn) (Wei Zhang)

**Figure S1. Chromosome-level genome assembly and karyotyping analysis of *Ne. cincticeps*, *Ne. nigropictus*, and *R. dorsalis*.**

(A) Comparison of genome assembly statistics among the three leafhopper species. (B) Contig contact matrices for the genomes of the three leafhopper species. Green borders delineate individual contigs, whereas blue borders delineate individual chromosomes. (C) Karyotype analysis of the three leafhopper species. Chromosomes were stained with carbolfuchsin and imaged by microscopy. All three species exhibit an XO sex-determination system, with males carrying a single X chromosome (XO) and females carrying two (XX). n represents the number of chromosome pairs. Bar = 5 µm.

**Figure S2.** **Distributions and gene structures of *CDCs* in the three leafhopper species.**

(A) Chromosomal distributions of the *CDCs*. Genomic and transcriptomic read coverage and gene structures are shown for the *CDCs* in *Ne. cincticeps* (B), and *Ne. nigropictus* (C). Read depths from genome resequencing and RNA sequencing data were log-transformed and plotted across the focal genomic regions, with numbers indicating the depth ranges. Splice junctions within CDSs and UTRs are indicated by solid and dotted red lines, respectively.

**Figure S3.** **Gene structures of *CDCs* in *R. dorsalis*.**

Genomic and transcriptomic read coverage and gene structures are shown for the *CDCs* in *R. dorsalis*. Read depths from genome resequencing and RNA sequencing data were log-transformed and plotted across the focal genomic regions, with numbers indicating the depth ranges. Splice junctions within CDSs and UTRs are indicated by solid and dotted red lines, respectively. The fragments used for PCR cloning of *RdCDC6* and *RdCDC9* are marked by red segments.

**Figure S4. Protein similarity clusters of the *MACPF*/*CDC* superfamily and the selection signals of leafhopper *CDCs*.**

The *pleurotolysin B* family, *MACPF* family, and *CDC* family clustered separately. The TCDB and UniProt accession numbers are listed for each of the proteins used.

**Figure S5.** **Phylogeny of *CDC* genes in the family Cicadellidae.**

(A) Species tree of Cicadellidae inferred from conserved single-copy genes, with *N. lugens* as the outgroup. The number of *CDC* genes in each species is shown on the right. (B) Maximum likelihood tree of all leafhopper *CDC* genes based on the protein sequence alignments. The circle indicates > 80% bootstrap support. Gene copies from the three focal species are highlighted. The *CDC* genes of *R. dorsalis* are divided into three clusters. The orthologs of *RdCDC9* are also present in other species, and their phylogenetic relationships are consistent with the species tree, suggesting that this gene copy originated in the ancestor of the lineage. Instead, the other two clusters represent species-specific new copies.

**Figure S6. Ka/Ks analysis of *CDC* genes in the family Cicadellidae.**

(A) Heatmap showing the pairwise Ka/Ks ratios between leafhopper *CDCs*. The topology relationships determined by the maximum likelihood method are shown above. The branches under positive selection (including *RdCDC4*, *RdCDC5*, *RdCDC6*, *RdCDC8*, *RdCDC9*, the ancestor branch of *NcCDC1* and *NnCDC1*, the ancestor of *NcCDC2* and *NnCDC2*, and the ancestor of *NcCDC3* and *NnCDC3*) are marked with a star and highlighted in red. These branches were detected by PAML analyses with the branch-site model. (B) Table summarizing the log-likelihood values under the null model (L0) and branch-site model (L1). Statistical significance was determined by a likelihood ratio test. The selected sites were analyzed using the Bayes empirical Bayes (BEB) method. * indicates *p* < 0.05.

**Figure S7. Sequence alignments of motifs related to cholesterol recognition and binding and protein structure predictions.**

(A) Amino acid sequence alignments of leafhopper and bacterial *CDCs* in conserved regions (loops L1, L2, and L3 and the undecapeptide). (B) The structures of leafhopper CDCs were predicted by AlphaFold 2 and aligned to perfringolysin O (PDB accession number: 1PFO). The structure of perfringolysin O is colored according to domain. Domain 1 (D1), red; D2, gray; D3, blue; D4, yellow; transmembrane hairpin 1 (TMH1), lemon; TMH2, purple; D4 loop 1 (L1), green; L2, orange; L3, cyan; and undecapeptide, pink.

**Figure S8. Determination of HEK293T and insect Sf9 cell viability.**

(A) Expression of RdCDC3-GFP, RdCDC6-GFP, and RdCDC9-GFP in Sf9 cells infected with recombinant baculoviruses, with GFP alone serving as the control. Images were acquired by confocal laser scanning microscopy using an excitation wavelength of 488 nm. Bar = 5 µm. (B) EC_50_ values derived from the MST data shown in Figure 3b. Data are presented as means ± SDs (*N* = 3). (C) Survival rates of *Bombyx mori* following injection with different concentrations of GST-RdCDC3, GST-RdCDC6, GST-RdCDC9, or GST. Eight silkworms were injected per concentration for each protein. Cell viability of HEK293T (D) and Sf9 (E) cells following exposure to RdCDC3, RdCDC6, or RdCDC9 for 24 h, as measured by MTT assays. (F) IC_50_ values of RdCDC3 in different cell lines. IC_50_ was defined as the protein concentration that reduced cell viability to 50%. (G) Images of HeLa cells preloaded with calcein and exposed to RdCDC3, RdCDC6 and RdCDC9 for 6 h. Bar = 20 µm.

**Figure S9. GUV assay of RdCDC3, 6, 9 and PLO.**

Representative time-lapse confocal images of GUVs containing 0% (A), 10% (C, G) or 20% (E) cholesterol following treatment with 400 nM RdCDC3 (top panel), RdCDC6 (middle panel), PLO (middle panel) or RdCDC9 (bottom panel). Time points are indicated on the top of the corresponding images. Magenta indicates FITC-dextran, whereas cyan, green, and yellow indicate rhodamine-PE incorporated into GUVs containing 0%, 10%, and 20% cholesterol, respectively. Bar = 10 μm. B, D, F, H. Statistical analysis of GUV perforation in A, C, E, G. Mean FITC-dextran intensities inside GUVs over time were normalized to FITC intensity in solution and plotted as a function of time.

**Figure S10.** ***RdCDC* RNAi in leafhoppers.**

(A‒B) RT‒qPCR detection of the expression of *RdCDC*3 and *RdCDC*9 in *RdCDC3* RNAi- or *RdCDC9* RNAi-treated insects; leafhoppers microinjected with *dsGFP* were used as controls. ***, *p* < 0.001; ns, not significant. *N* = 18. (C‒D) Detection of the RdCDC3 and RdCDC9 proteins in *RdCDC3* RNAi- or *RdCDC9* RNAi-treated insects via western blotting with antibodies against RdCDC3 or RdCDC9.

**Figure S11.** **The diversity and function of the leafhopper gut microbiome.**

(A) Absolute abundances of identified and undetermined taxa across different samples. (B) Box plots showing the Shannon, Simpson, Chao1, ACE (abundance-based coverage estimator) indices of bacterial communities in the *dsGFP* and *dsRdCDC9* groups. No significant difference was detected between groups (Wilcoxon test). (C) The PCoA results based on the Bray-Curtis distance matrix, which is calculated from the abundance profiles of bacteria OTUs. Ellipses indicate 50% confidence intervals. PCoA1 and PCoA2 explain 75.6% and 20.8% of the variance, respectively. (D) Cladogram showing the phylogenetic relationships of differentially abundant taxa between the two groups. Red and gray circles indicate taxa enriched in the *dsRdCDC9* and *dsGFP* samples (LDA score > 2, *p* < 0.05), respectively. The concentric circles in the diagram, from inner to outer, sequentially represent the taxa at the level of phylum, class, order, family, genus, and species. Supraspecific taxonomic information is listed below the diagram. (E) Bar plot showing the LDA score of differentially abundant taxa in the *dsRdCDC9* (red) and *dsGFP* samples (gray) in the LEfSe analysis (LDA score > 2, *p* < 0.05). (F) Absolute abundances of genes assigned to each category in the VFDB across samples. (G) Heatmap showing the pair-wise similarities (1 - Bray-Curtis distance) across samples. The distance is calculated from the abundance profiles of VFDB categories. G, *dsGFP*. C9, *dsRdCDC9*. *N* = 4 per group. (H) Heatmap showing the scaled abundance of VF genes across samples. Only differentially abundant genes are shown (Wilcoxon test, *p* < 0.05).
